# Supplementary material for: Sarcomeres Morphology and Z-Line Arrangement Disarray Induced by Ventricular Premature Contractions through the Rac2/Cofilin Pathway
Source: Int J Mol Sci. 2021 Oct 18;22(20):11244. doi: 10.3390/ijms222011244 (PMC8541677; doi:10.3390/ijms222011244)

Content

Supplemental Table S1. 1

Supplemental Table S2. 10

Supplemental Table S3. 24

Supplemental Table S4. 37

Supplemental Table S5. 45

Supplemental Figure S1. 46

Supplemental Figure S2. 47

Supplemental Figure S3. 49

Supplemental Figure S4. 51

Supplemental Figure S5. 52

Supplemental Table S1.

Selected signature Sus Scrofa gene expressions altered > 1.5-fold change (Log_2FC (RVOT-VPC/sham control) > 0.58) in the left ventricular septal tissues of RVOT VPC group vs. sham control group at 6-month follow-up

Symbol	Description	Log_2 FC	P value
gene:ENSSSCG00000038101		6.40	0.01
gene:ENSSSCG00000025086		5.67	0.03
UMODL1	uromodulin like 1	5.45	0.00
MDGA2	MAM domain containing glycosylphosphatidylinositol anchor 2	5.09	0.05
ADIPOQ	adiponectin, C1Q and collagen domain containing	4.89	0.00
CTNND2	catenin delta 2	4.53	0.00
SCN3B	sodium voltage-gated channel beta subunit 3	4.40	0.00
UBXN10	UBX domain protein 10	4.00	0.00
OAZ3	ornithine decarboxylase antizyme 3	3.88	0.00
SYT1	synaptotagmin 1	3.80	0.00
PLIN1	perilipin 1	3.79	0.03
SLN	sarcolipin	3.28	0.00
gene:ENSSSCG00000034962		3.25	0.00
IFITM5	interferon induced transmembrane protein 5	3.17	0.04
NPAS4	neuronal PAS domain protein 4	3.11	0.04
VSIG2	V-set and immunoglobulin domain containing 2	3.07	0.00
C1orf210	chromosome 1 open reading frame 210	3.07	0.00
RBP7	retinol binding protein 7	2.94	0.01
gene:ENSSSCG00000001395		2.94	0.00
KCNG2	potassium voltage-gated channel modifier subfamily G member 2	2.84	0.01
gene:ENSSSCG00000011324		2.76	0.00
IL23A	interleukin 23 subunit alpha	2.75	0.02
gene:ENSSSCG00000036748		2.75	0.02
gene:ENSSSCG00000006525		2.74	0.05
gene:ENSSSCG00000040285		2.60	0.01
NNAT	neuronatin	2.59	0.00
KRT18	keratin 18	2.59	0.02

FGF16	fibroblast growth factor 16	2.58	0.00
gene:ENSSSCG00000031997		2.58	0.00
DLK2	delta like non-canonical Notch ligand 2	2.55	0.04
TNNC2	troponin C2, fast skeletal type	2.48	0.01
EYA1	EYA transcriptional coactivator and phosphatase 1	2.47	0.00
FOSB	FosB proto-oncogene, AP-1 transcription factor subunit	2.44	0.00
BDNF	brain derived neurotrophic factor	2.44	0.00
gene:ENSSSCG00000014186		2.39	0.02
gene:ENSSSCG00000036109		2.38	0.02
DNAAF1	dynein axonemal assembly factor 1	2.35	0.04
SPOCK3	SPARC/osteonectin, cwcv and kazal like domains proteoglycan 3	2.35	0.02
RGS9BP	regulator of G protein signaling 9 binding protein	2.31	0.02
EMID1	EMI domain containing 1	2.29	0.01
PLEK2	pleckstrin 2	2.24	0.00
gene:ENSSSCG00000031255		2.24	0.01
PTPN18	protein tyrosine phosphatase, non-receptor type 18	2.18	0.01
SSUH2	ssu-2 homolog (C. elegans)	2.14	0.03
CDH12	cadherin 12	2.13	0.02
IGSF5	immunoglobulin superfamily member 5	2.09	0.03
SUSD4	sushi domain containing 4	2.08	0.02
B4GALT6	beta-1,4-galactosyltransferase 6	2.08	0.02
gene:ENSSSCG00000040146		2.08	0.01
LONRF2	LON peptidase N-terminal domain and ring finger 2	2.02	0.02
SMCO1	single-pass membrane protein with coiled-coil domains 1	2.01	0.01
NPPA	natriuretic peptide A	1.98	0.00
ARG2	arginase 2	1.93	0.00
TMEM82	transmembrane protein 82	1.93	0.05
TMEM163	transmembrane protein 163	1.91	0.01
PPEF2	protein phosphatase with EF-hand domain 2	1.90	0.02
IL20RA	interleukin 20 receptor subunit alpha	1.88	0.04

LIPN	lipase family member N	1.87	0.04
MYMK	myomaker, myoblast fusion factor	1.87	0.00
gene:ENSSSCG00000040453		1.86	0.00
ALDOB	aldolase, fructose-bisphosphate B	1.86	0.00
NR0B2	nuclear receptor subfamily 0 group B member 2	1.85	0.00
gene:ENSSSCG00000014272		1.82	0.00
CYP2B22		1.82	0.03
SHOX2	short stature homeobox 2	1.78	0.01
SLC39A12	solute carrier family 39 member 12	1.78	0.03
FRZB	frizzled related protein	1.78	0.01
IRX2	iroquois homeobox 2	1.75	0.01
gene:ENSSSCG00000005479		1.74	0.05
gene:ENSSSCG00000035477		1.72	0.04
AGXT2	alanine--glyoxylate aminotransferase 2	1.72	0.05
DKK3	dickkopf WNT signaling pathway inhibitor 3	1.72	0.00
MNS1	meiosis specific nuclear structural 1	1.71	0.00
gene:ENSSSCG00000029866		1.71	0.00
IQCF2	IQ motif containing F2	1.71	0.03
CHI3L2	chitinase 3 like 2	1.71	0.03
gene:ENSSSCG00000014975		1.71	0.03
gene:ENSSSCG00000009500		1.70	0.00
gene:ENSSSCG00000028944		1.68	0.01
CENPS	centromere protein S	1.68	0.02
VSNL1	visinin like 1	1.68	0.04
SCIN	scinderin	1.66	0.04
STX5	syntaxin 5	1.65	0.03
gene:ENSSSCG00000036911		1.63	0.02
GRIK4	glutamate ionotropic receptor kainate type subunit 4	1.63	0.03
GRAMD1B	GRAM domain containing 1B	1.62	0.00
PADI2	peptidyl arginine deiminase 2	1.60	0.00
GADL1	glutamate decarboxylase like 1	1.59	0.00
L3HYPDH	trans-L-3-hydroxyproline dehydratase	1.59	0.00
ITGAE	integrin subunit alpha E	1.57	0.00
LRRC23	leucine rich repeat containing 23	1.55	0.05
OSR2	odd-skipped related transciption factor 2	1.55	0.04

EXD1	exonuclease 3'-5' domain containing 1	1.54	0.00
HMGCLL1	3-hydroxymethyl-3-methylglutaryl-CoA lyase like 1	1.53	0.01
IRX6	iroquois homeobox 6	1.52	0.00
KCNS3	potassium voltage-gated channel modifier subfamily S member 3	1.51	0.01
NRSN2	neurensin 2	1.49	0.02
PLIN5	perilipin 5	1.48	0.00
UOX	urate oxidase (pseudogene)	1.46	0.04
GNB3	G protein subunit beta 3	1.46	0.00
WWC1	WW and C2 domain containing 1	1.44	0.02
MYOZ1	myozenin 1	1.43	0.03
HBB	hemoglobin subunit beta	1.42	0.01
SERPINF1	serpin family F member 1	1.38	0.01
HEPACAM	hepatic and glial cell adhesion molecule	1.38	0.01
GLIPR1	GLI pathogenesis related 1	1.37	0.00
SAYSD1	SAYSVFN motif domain containing 1	1.37	0.02
RGS6	regulator of G protein signaling 6	1.36	0.02
gene:ENSSSCG00000013593		1.36	0.02
ENPP5	ectonucleotide pyrophosphatase/phosphodiesterase 5 (putative)	1.34	0.01
gene:ENSSSCG00000035706		1.33	0.05
CCDC136	coiled-coil domain containing 136	1.33	0.00
TMCO4	transmembrane and coiled-coil domains 4	1.33	0.02
STRC	stereocilin	1.31	0.01
gene:ENSSSCG00000038945		1.31	0.02
ATF3	activating transcription factor 3	1.30	0.00
LRRC4B	leucine rich repeat containing 4B	1.30	0.01
NEU3	neuraminidase 3	1.29	0.00
NR4A1	nuclear receptor subfamily 4 group A member 1	1.27	0.00
ADRA2B	adrenoceptor alpha 2B	1.27	0.00
ACSM3	acyl-CoA synthetase medium chain family member 3	1.26	0.03
CCDC173	coiled-coil domain containing 173	1.25	0.02
XKRX	XK related, X-linked	1.24	0.01
NAAA	N-acylethanolamine acid amidase	1.24	0.00

ADAMTS14	ADAM metallopeptidase with thrombospondin type 1 motif 14	1.23	0.02
gene:ENSSSCG00000016215		1.23	0.02
IL10RA	interleukin 10 receptor subunit alpha	1.23	0.00
FAM155B	family with sequence similarity 155 member B	1.22	0.00
RNF180	ring finger protein 180	1.22	0.00
DUSP5	dual specificity phosphatase 5	1.21	0.03
TNNT1	troponin T1, slow skeletal type	1.21	0.01
RIPOR2	RHO family interacting cell polarization regulator 2	1.21	0.00
FGD6	FYVE, RhoGEF and PH domain containing 6	1.20	0.00
FGFRL1	fibroblast growth factor receptor like 1	1.19	0.01
EFCC1	EF-hand and coiled-coil domain containing 1	1.19	0.03
IFI27L2	interferon alpha inducible protein 27 like 2	1.18	0.04
RAB6B	RAB6B, member RAS oncogene family	1.18	0.05
FRMD1	FERM domain containing 1	1.17	0.03
AGT	angiotensinogen	1.16	0.01
DRD2	dopamine receptor D2	1.16	0.04
HIST1H2BD	histone cluster 1 H2B family member d	1.16	0.00
GABRA4	gamma-aminobutyric acid type A receptor alpha4 subunit	1.16	0.00
FAM46B	family with sequence similarity 46 member B	1.15	0.00
TSPAN2	tetraspanin 2	1.14	0.01
gene:ENSSSCG00000031450		1.12	0.00
CXCL2	C-X-C motif chemokine ligand 2	1.09	0.02
TACR1	tachykinin receptor 1	1.09	0.04
GPC3	glypican 3	1.09	0.00
gene:ENSSSCG00000003793		1.09	0.04
ADIRF	adipogenesis regulatory factor	1.08	0.02
PRMT8	protein arginine methyltransferase 8	1.07	0.01
gene:ENSSSCG00000011147		1.05	0.00
MYO5B	myosin VB	1.05	0.05
ZPBP	zona pellucida binding protein	1.03	0.04
EDIL3	EGF like repeats and discoidin domains 3	1.02	0.00

FADS3	fatty acid desaturase 3	1.00	0.00
THRSP	thyroid hormone responsive	1.00	0.03
CSDC2	cold shock domain containing C2	0.99	0.05
KIAA1324L	KIAA1324 like	0.98	0.00
OLFM1	olfactomedin 1	0.96	0.04
S1PR2	sphingosine-1-phosphate receptor 2	0.96	0.01
GCK	glucokinase	0.95	0.04
ACER1	alkaline ceramidase 1	0.94	0.04
WSCD2	WSC domain containing 2	0.94	0.01
MLLT11	MLLT11, transcription factor 7 cofactor	0.94	0.00
NR4A3	nuclear receptor subfamily 4 group A member 3	0.93	0.01
SORL1	sortilin related receptor 1	0.93	0.00
CYB5A	cytochrome b5 type A	0.91	0.00
RASD1	ras related dexamethasone induced 1	0.90	0.04
GNMT	glycine N-methyltransferase	0.89	0.02
IGF2BP3	insulin like growth factor 2 mRNA binding protein 3	0.89	0.04
gene:ENSSSCG00000036306		0.89	0.04
FAM46C	family with sequence similarity 46 member C	0.89	0.04
MBOAT2	membrane bound O-acyltransferase domain containing 2	0.87	0.01
IER5	immediate early response 5	0.87	0.01
gene:ENSSSCG00000010278		0.87	0.00
FBLN5	fibulin 5	0.86	0.03
CDKL1	cyclin dependent kinase like 1	0.86	0.04
PIM1	Pim-1 proto-oncogene, serine/threonine kinase	0.86	0.03
ASB5	ankyrin repeat and SOCS box containing 5	0.86	0.00
CYFIP2	cytoplasmic FMR1 interacting protein 2	0.86	0.02
CCSAP	centriole, cilia and spindle associated protein	0.85	0.02
MGARP	mitochondria localized glutamic acid rich protein	0.85	0.00
RERG	RAS like estrogen regulated growth inhibitor	0.85	0.03
gene:ENSSSCG00000008981		0.84	0.01

RAB9B	RAB9B, member RAS oncogene family	0.84	0.01
PLPP4	phospholipid phosphatase 4	0.84	0.02
PDIA2	protein disulfide isomerase family A member 2	0.83	0.02
gene:ENSSSCG00000034689		0.82	0.02
gene:ENSSSCG00000003451		0.82	0.03
SDR39U1	short chain dehydrogenase/reductase family 39U member 1	0.82	0.01
PAK1	p21 (RAC1) activated kinase 1	0.82	0.04
BTG2	BTG anti-proliferation factor 2	0.81	0.05
FABP3	fatty acid binding protein 3	0.81	0.00
ADRB2	adrenoceptor beta 2	0.80	0.03
GPC4	glypican 4	0.80	0.01
TWF2	twinfilin actin binding protein 2	0.80	0.00
TCF21	transcription factor 21	0.80	0.00
gene:ENSSSCG00000035908		0.80	0.01
VIPR2	vasoactive intestinal peptide receptor 2	0.79	0.04
MYLK	myosin light chain kinase	0.79	0.02
PIK3R6	phosphoinositide-3-kinase regulatory subunit 6	0.79	0.02
LGALS1	galectin 1	0.78	0.00
C12orf75	chromosome 12 open reading frame 75	0.78	0.03
EFHC1	EF-hand domain containing 1	0.78	0.04
GGA2	golgi associated, gamma adaptin ear containing, ARF binding protein 2	0.78	0.00
SYNC	syncoilin, intermediate filament protein	0.77	0.00
GRAMD1C	GRAM domain containing 1C	0.77	0.05
TSPAN9	tetraspanin 9	0.77	0.00
KLHL32	kelch like family member 32	0.77	0.02
CXXC5	CXXC finger protein 5	0.76	0.04
NR4A2	nuclear receptor subfamily 4 group A member 2	0.76	0.01
FRMD5	FERM domain containing 5	0.76	0.02
DCBLD2	discoidin, CUB and LCCL domain containing 2	0.76	0.03
CDON	cell adhesion associated, oncogene regulated	0.75	0.02
CSRNP1	cysteine and serine rich nuclear protein 1	0.75	0.03

ABCC9	ATP binding cassette subfamily C member 9	0.75	0.00
ASPN	asporin	0.75	0.05
gene:ENSSSCG00000035424		0.74	0.00
EPB41L5	erythrocyte membrane protein band 4.1 like 5	0.74	0.01
ELL3	elongation factor for RNA polymerase II 3	0.72	0.04
TINAGL1	tubulointerstitial nephritis antigen like 1	0.72	0.00
RTN4RL1	reticulon 4 receptor like 1	0.72	0.02
DHRS4	dehydrogenase/reductase 4	0.72	0.00
CDKN2C	cyclin dependent kinase inhibitor 2C	0.71	0.03
SPCS3	signal peptidase complex subunit 3	0.71	0.01
LDHB	lactate dehydrogenase B	0.71	0.00
SLC25A33	solute carrier family 25 member 33	0.71	0.03
HDHD3	haloacid dehalogenase like hydrolase domain containing 3	0.70	0.05
FAM81A	family with sequence similarity 81 member A	0.70	0.02
ATP13A3	ATPase 13A3	0.69	0.00
TCIM	transcriptional and immune response regulator	0.69	0.02
F3	coagulation factor III, tissue factor	0.69	0.02
KLF4	Kruppel like factor 4	0.69	0.01
AGA	aspartylglucosaminidase	0.69	0.01
C22orf23	chromosome 22 open reading frame 23	0.68	0.03
gene:ENSSSCG00000005623		0.67	0.02
ETV1	ETS variant 1	0.66	0.05
CTSC	cathepsin C	0.66	0.00
TNIK	TRAF2 and NCK interacting kinase	0.66	0.03
ALDH5A1	aldehyde dehydrogenase 5 family member A1	0.66	0.01
MXRA7	matrix remodeling associated 7	0.66	0.03
HEBP1	heme binding protein 1	0.66	0.03
PQLC1	PQ loop repeat containing 1	0.66	0.01
gene:ENSSSCG00000027013		0.66	0.04
SERTAD3	SERTA domain containing 3	0.65	0.05
CASQ1	calsequestrin 1	0.65	0.03
ARHGAP18	Rho GTPase activating protein 18	0.65	0.01

KLF10	Kruppel like factor 10	0.64	0.04
SLC29A1	solute carrier family 29 member 1 (Augustine blood group)	0.64	0.00
FBLIM1	filamin binding LIM protein 1	0.64	0.04
HECW2	HECT, C2 and WW domain containing E3 ubiquitin protein ligase 2	0.63	0.03
gene:ENSSSCG00000007749		0.63	0.03
ARMC6	armadillo repeat containing 6	0.63	0.03
PHF24	PHD finger protein 24	0.62	0.04
PAH	phenylalanine hydroxylase	0.62	0.03
ANTXR2	anthrax toxin receptor 2	0.62	0.00
NIM1K	NIM1 serine/threonine protein kinase	0.61	0.03
PPP1R3B	protein phosphatase 1 regulatory subunit 3B	0.61	0.04
OMD	osteomodulin	0.61	0.01
LRRC10	leucine rich repeat containing 10	0.61	0.04
SERPINB2	serpin family B member 2	0.60	0.01
CCNT1	cyclin T1	0.60	0.00
RHOBTB3	Rho related BTB domain containing 3	0.60	0.02
IGSF1	immunoglobulin superfamily member 1	0.59	0.01
SLCO3A1	solute carrier organic anion transporter family member 3A1	0.59	0.02
OLFML2A	olfactomedin like 2A	0.59	0.02
gene:ENSSSCG00000031893		0.59	0.01
LAMC2	laminin subunit gamma 2	0.59	0.04

Supplemental Table S2.

Selected signature Sus Scrofa gene expressions altered < 0.66-fold change (Log_2FC (RVOT-VPC/sham control) < −0.58) in the left ventricular septal tissues of RVOT VPC group vs. sham control group at 6-month follow-up

Symbol	Description	Log_2 FC	P value
CHI3L1	chitinase 3 like 1	-8.03	0.00
gene:ENSSSCG00000034356		-7.98	0.00
CCL20	C-C motif chemokine ligand 20	-7.63	0.00
HYAL3	hyaluronoglucosaminidase 3	-7.60	0.00
gene:ENSSSCG00000039574		-6.68	0.01
LIPM	lipase family member M	-6.51	0.00
CDHR1	cadherin related family member 1	-5.96	0.00
gene:ENSSSCG00000039803		-5.88	0.02
gene:ENSSSCG00000037349		-5.82	0.02
gene:ENSSSCG00000020695		-5.51	0.00
RLBP1	retinaldehyde binding protein 1	-5.37	0.00
TNC	tenascin C	-4.61	0.00
CAPN6	calpain 6	-4.58	0.00
COMP	cartilage oligomeric matrix protein	-4.58	0.01
MYBPH	myosin binding protein H	-4.44	0.00
MT1A	metallothionein 1A	-4.40	0.00
PRSS35	protease, serine 35	-4.32	0.00
CACNA1S	calcium voltage-gated channel subunit alpha1 S	-4.15	0.00
gene:ENSSSCG00000036445		-4.01	0.00
CTHRC1	collagen triple helix repeat containing 1	-3.94	0.00
FASLG	Fas ligand	-3.91	0.00
ISLR2	immunoglobulin superfamily containing leucine rich repeat 2	-3.89	0.01
NALCN	sodium leak channel, non-selective	-3.79	0.00
S100A8	S100 calcium binding protein A8	-3.75	0.00

gene:ENSSSCG00000037358		-3.75	0.00
DEPDC1	DEP domain containing 1	-3.74	0.02
gene:ENSSSCG00000040236		-3.59	0.03
MELK	maternal embryonic leucine zipper kinase	-3.58	0.02
gene:ENSSSCG00000024911		-3.49	0.02
gene:ENSSSCG00000029449		-3.43	0.01
NKAIN3	sodium/potassium transporting ATPase interacting 3	-3.40	0.03
CPA1	carboxypeptidase A1	-3.38	0.00
SLC9A3	solute carrier family 9 member A3	-3.36	0.00
CENPA	centromere protein A	-3.31	0.03
gene:ENSSSCG00000006376		-3.29	0.02
ANKRD2	ankyrin repeat domain 2	-3.28	0.00
CDCA8	cell division cycle associated 8	-3.23	0.00
MATN4	matrilin 4	-3.23	0.00
GPRL15		-3.22	0.03
FAM83B	family with sequence similarity 83 member B	-3.19	0.03
CYP3A39		-3.16	0.00
TSHR	thyroid stimulating hormone receptor	-3.11	0.01
S100A9	S100 calcium binding protein A9	-3.10	0.00
MMP8	matrix metallopeptidase 8	-3.09	0.04
GKN2	gastrokine 2	-2.94	0.03
FREM1	FRAS1 related extracellular matrix 1	-2.93	0.01
S100A12	S100 calcium binding protein A12	-2.92	0.00
gene:ENSSSCG00000015839		-2.85	0.03
ASF1B	anti-silencing function 1B histone chaperone	-2.85	0.02
DIAPH3	diaphanous related formin 3	-2.85	0.03
CDT1	chromatin licensing and DNA replication factor 1	-2.83	0.02

RELL2	RELT like 2	-2.82	0.03
gene:ENSSSCG00000036586		-2.79	0.04
TAC4	tachykinin 4	-2.79	0.04
FADS6	fatty acid desaturase 6	-2.78	0.01
gene:ENSSSCG00000038068		-2.77	0.00
POSTN	periostin	-2.74	0.00
TGM3	transglutaminase 3	-2.69	0.00
gene:ENSSSCG00000002749		-2.69	0.00
CDC6	cell division cycle 6	-2.68	0.01
AMCF-II		-2.66	0.02
TMIGD1	transmembrane and immunoglobulin domain containing 1	-2.66	0.03
ANPEP	alanyl aminopeptidase, membrane	-2.60	0.00
CRLF1	cytokine receptor like factor 1	-2.57	0.00
gene:ENSSSCG00000033958		-2.55	0.03
CENPL	centromere protein L	-2.55	0.05
FOXH1	forkhead box H1	-2.49	0.02
OLFM3	olfactomedin 3	-2.49	0.05
SELP	selectin P	-2.48	0.00
C6	complement C6	-2.47	0.04
PI15	peptidase inhibitor 15	-2.42	0.00
CDA	cytidine deaminase	-2.42	0.01
TOX	thymocyte selection associated high mobility group box	-2.40	0.02
gene:ENSSSCG00000031037		-2.40	0.01
CEMIP	cell migration inducing hyaluronan binding protein	-2.37	0.04
RASSF10	Ras association domain family member 10	-2.34	0.03
CENPT	centromere protein T	-2.33	0.02
gene:ENSSSCG00000009347		-2.33	0.03
CCL11	C-C motif chemokine ligand 11	-2.31	0.01

gene:ENSSSCG00000031292		-2.30	0.04
COL12A1	collagen type XII alpha 1 chain	-2.29	0.00
gene:ENSSSCG00000039222		-2.29	0.01
C16orf54	chromosome 16 open reading frame 54	-2.29	0.05
FAM180B	family with sequence similarity 180 member B	-2.29	0.05
NLRP13	NLR family pyrin domain containing 13	-2.28	0.00
AMBN	ameloblastin	-2.28	0.05
TMEM59L	transmembrane protein 59 like	-2.27	0.01
TLR8	toll like receptor 8	-2.26	0.01
TOP2A	DNA topoisomerase II alpha	-2.25	0.00
TIMP1	TIMP metallopeptidase inhibitor 1	-2.24	0.00
DLGAP5	DLG associated protein 5	-2.23	0.02
MMRN2	multimerin 2	-2.22	0.00
INHBA	inhibin beta A subunit	-2.21	0.00
MSTN	myostatin	-2.20	0.01
ITGBL1	integrin subunit beta like 1	-2.19	0.02
OLFML2B	olfactomedin like 2B	-2.17	0.00
gene:ENSSSCG00000031741		-2.16	0.01
gene:ENSSSCG00000038557		-2.15	0.00
SERTAD4	SERTA domain containing 4	-2.14	0.00
WNT2	Wnt family member 2	-2.14	0.03
ACTG2	actin, gamma 2, smooth muscle, enteric	-2.11	0.00
KIF11	kinesin family member 11	-2.11	0.01
MMP9	matrix metallopeptidase 9	-2.10	0.03
FN1	fibronectin 1	-2.10	0.00
CP	ceruloplasmin	-2.09	0.01
ASPM	abnormal spindle microtubule assembly	-2.08	0.04
VWA5B2	von Willebrand factor A domain containing 5B2	-2.08	0.01
CLCA2	chloride channel accessory 2	-2.06	0.04
BUB1B	BUB1 mitotic checkpoint serine/threonine kinase B	-2.05	0.04
gene:ENSSSCG0000002225		-2.03	0.04

8			
gene:ENSSSCG00000029414		-2.03	0.01
CENPF	centromere protein F	-2.03	0.01
TREML2	triggering receptor expressed on myeloid cells like 2	-2.02	0.02
HDC	histidine decarboxylase	-2.01	0.04
gene:ENSSSCG00000040350		-2.01	0.00
ADAM12	ADAM metallopeptidase domain 12	-2.00	0.02
HIVEP3	human immunodeficiency virus type I enhancer binding protein 3	-2.00	0.00
gene:ENSSSCG00000026302		-1.99	0.00
gene:ENSSSCG00000030469		-1.99	0.04
LYZ	lysozyme	-1.98	0.00
CDCA7L	cell division cycle associated 7 like	-1.98	0.00
gene:ENSSSCG00000023322		-1.96	0.01
GBX1	gastrulation brain homeobox 1	-1.96	0.04
KCNJ5	potassium voltage-gated channel subfamily J member 5	-1.94	0.00
gene:ENSSSCG00000002849		-1.94	0.02
HTR2B	5-hydroxytryptamine receptor 2B	-1.93	0.04
CPXM1	carboxypeptidase X, M14 family member 1	-1.92	0.00
FGFR2	fibroblast growth factor receptor 2	-1.91	0.01
TBC1D10C	TBC1 domain family member 10C	-1.91	0.00
gene:ENSSSCG00000037214		-1.89	0.02
BAALC	BAALC, MAP3K1 and KLF4 binding	-1.88	0.00
gene:ENSSSCG00000013788		-1.86	0.00
CDCA5	cell division cycle associated 5	-1.85	0.02
PVRIG	PVR related immunoglobulin domain containing	-1.85	0.04

gene:ENSSSCG00000030300		-1.85	0.00
UBE2C	ubiquitin conjugating enzyme E2 C	-1.81	0.03
CD69	CD69 molecule	-1.80	0.00
DDAH1	dimethylarginine dimethylaminohydrolase 1	-1.80	0.00
MEGF11	multiple EGF like domains 11	-1.80	0.03
gene:ENSSSCG00000016451		-1.79	0.01
NLRP11	NLR family pyrin domain containing 11	-1.78	0.02
LIF	LIF, interleukin 6 family cytokine	-1.77	0.04
gene:ENSSSCG00000020967		-1.69	0.02
ANGPTL7	angiopoietin like 7	-1.68	0.05
gene:ENSSSCG00000028695		-1.67	0.03
CX3CR1	C-X3-C motif chemokine receptor 1	-1.67	0.02
CHRNA4	cholinergic receptor nicotinic alpha 4 subunit	-1.67	0.02
FAM198A	family with sequence similarity 198 member A	-1.66	0.02
SLC11A1	solute carrier family 11 member 1	-1.66	0.04
SH2D1A	SH2 domain containing 1A	-1.64	0.04
GZMA_2		-1.64	0.04
GFRA3	GDNF family receptor alpha 3	-1.62	0.01
gene:ENSSSCG00000012733		-1.62	0.02
VCAN	versican	-1.62	0.00
COL6A6	collagen type VI alpha 6 chain	-1.62	0.00
DNAJC6	DnaJ heat shock protein family (Hsp40) member C6	-1.61	0.01
ATP8	ATP synthase F0 subunit 8	-1.61	0.04
gene:ENSSSCG00000021899		-1.60	0.02
TNFRSF11B	TNF receptor superfamily member 11b	-1.58	0.04
COL8A1	collagen type VIII alpha 1 chain	-1.58	0.00
HGF	hepatocyte growth factor	-1.57	0.01
gene:ENSSSCG0000001021		-1.57	0.04

4			
gene:ENSSSCG00000036822		-1.55	0.03
PYGO1	pygopus family PHD finger 1	-1.55	0.03
MRAP2	melanocortin 2 receptor accessory protein 2	-1.55	0.05
MT3	metallothionein 3	-1.55	0.00
GLRB	glycine receptor beta	-1.54	0.05
gene:ENSSSCG00000039107		-1.54	0.04
gene:ENSSSCG00000036348		-1.54	0.04
gene:ENSSSCG00000026653		-1.54	0.02
PANX1	pannexin 1	-1.52	0.02
PHGDH	phosphoglycerate dehydrogenase	-1.51	0.02
MYO1G	myosin IG	-1.51	0.01
gene:ENSSSCG00000034853		-1.51	0.00
gene:ENSSSCG00000010948		-1.50	0.02
gene:ENSSSCG00000028341		-1.50	0.00
SDC1	syndecan 1	-1.50	0.03
BGN	biglycan	-1.48	0.00
CLEC11A	C-type lectin domain containing 11A	-1.46	0.05
CCL5	C-C motif chemokine ligand 5	-1.46	0.01
TCF7	transcription factor 7	-1.45	0.04
CDH24	cadherin 24	-1.45	0.00
gene:ENSSSCG00000009213		-1.44	0.00
PHLDB3	pleckstrin homology like domain family B member 3	-1.43	0.03
SLC9A3R1	SLC9A3 regulator 1	-1.42	0.00
MSC	musculin	-1.42	0.01
C15orf48	chromosome 15 open reading frame 48	-1.41	0.04
IGF1	insulin like growth factor 1	-1.39	0.02
TJP3	tight junction protein 3	-1.38	0.03

IQGAP3	IQ motif containing GTPase activating protein 3	-1.37	0.05
B3GNT8	UDP-GlcNAc:betaGal beta-1,3-N-acetylglucosaminyltransferase 8	-1.37	0.03
PRRX1	paired related homeobox 1	-1.37	0.00
FAM43B	family with sequence similarity 43 member B	-1.36	0.04
COL3A1	collagen type III alpha 1 chain	-1.36	0.00
SLPI	secretory leukocyte peptidase inhibitor	-1.36	0.00
PLCB2	phospholipase C beta 2	-1.34	0.02
LPXN	leupaxin	-1.33	0.05
MBP	myelin basic protein	-1.32	0.02
COL1A1	collagen type I alpha 1 chain	-1.32	0.00
NEB	nebulin	-1.32	0.05
F13A1	coagulation factor XIII A chain	-1.32	0.01
GIMAP8	GTPase, IMAP family member 8	-1.30	0.03
gene:ENSSSCG00000008898		-1.30	0.00
ALDH1A3	aldehyde dehydrogenase 1 family member A3	-1.29	0.01
PCSK5	proprotein convertase subtilisin/kexin type 5	-1.28	0.01
ITGA4	integrin subunit alpha 4	-1.28	0.01
PAK3	p21 (RAC1) activated kinase 3	-1.28	0.04
CCR1	C-C motif chemokine receptor 1	-1.27	0.04
SESN2	sestrin 2	-1.27	0.01
NTRK2	neurotrophic receptor tyrosine kinase 2	-1.26	0.04
GNLY	granulysin	-1.26	0.00
INPP1	inositol polyphosphate-1-phosphatase	-1.25	0.00
CD3E	CD3e molecule	-1.24	0.02
RUNX1	runt related transcription factor 1	-1.23	0.03
DPH1	diphthamide biosynthesis 1	-1.23	0.01
CCDC80	coiled-coil domain containing 80	-1.22	0.00
ZNF683	zinc finger protein 683	-1.20	0.03
ZNF503	zinc finger protein 503	-1.20	0.00
DNAH8	dynein axonemal heavy chain 8	-1.19	0.01
LAX1	lymphocyte transmembrane adaptor 1	-1.19	0.05

gene:ENSSSCG00000029696		-1.18	0.01
gene:ENSSSCG00000004830		-1.14	0.02
SERPINE2	serpin family E member 2	-1.14	0.05
CAPG	capping actin protein, gelsolin like	-1.14	0.04
THEMIS2	thymocyte selection associated family member 2	-1.14	0.03
PLEKHB1	pleckstrin homology domain containing B1	-1.13	0.04
PTGER2	prostaglandin E receptor 2	-1.13	0.00
GALNT16	polypeptide N-acetylgalactosaminyltransferase 16	-1.11	0.02
ABI3BP	ABI family member 3 binding protein	-1.11	0.01
S100A6	S100 calcium binding protein A6	-1.10	0.01
GIMAP7	GTPase, IMAP family member 7	-1.09	0.03
gene:ENSSSCG00000013434		-1.08	0.04
COL1A2	collagen type I alpha 2 chain	-1.08	0.01
SLCO2A1	solute carrier organic anion transporter family member 2A1	-1.05	0.00
MYOF	myoferlin	-1.05	0.00
CD8A	CD8a molecule	-1.03	0.05
KIF21B	kinesin family member 21B	-1.03	0.04
CD52	CD52 molecule	-1.03	0.05
ARHGAP4	Rho GTPase activating protein 4	-1.02	0.02
UBASH3B	ubiquitin associated and SH3 domain containing B	-1.01	0.01
gene:ENSSSCG00000035256		-1.01	0.02
gene:ENSSSCG00000027826		-1.00	0.01
TTC38	tetratricopeptide repeat domain 38	-0.99	0.03
FMOD	fibromodulin	-0.98	0.04
PLVAP	plasmalemma vesicle associated protein	-0.98	0.00
PPIC	peptidylprolyl isomerase C	-0.97	0.02
gene:ENSSSCG00000033286		-0.97	0.04

FUT4	fucosyltransferase 4	-0.97	0.05
THPO	thrombopoietin	-0.97	0.02
PLEKHA6	pleckstrin homology domain containing A6	-0.96	0.00
PTPRC	protein tyrosine phosphatase, receptor type C	-0.95	0.01
COL6A3	collagen type VI alpha 3 chain	-0.95	0.00
SFRP4	secreted frizzled related protein 4	-0.94	0.05
FBF1	Fas binding factor 1	-0.93	0.00
DOCK8	dedicator of cytokinesis 8	-0.93	0.04
KIAA1211	KIAA1211	-0.92	0.03
gene:ENSSSCG00000033089		-0.92	0.03
PI16	peptidase inhibitor 16	-0.91	0.02
CD109	CD109 molecule	-0.91	0.05
IGFBP2	insulin like growth factor binding protein 2	-0.90	0.05
PIEZO1	piezo type mechanosensitive ion channel component 1	-0.90	0.01
COL14A1	collagen type XIV alpha 1 chain	-0.90	0.02
PMEPA1	prostate transmembrane protein, androgen induced 1	-0.90	0.01
NHSL1	NHS like 1	-0.90	0.01
LPCAT4	lysophosphatidylcholine acyltransferase 4	-0.89	0.00
THBS3	thrombospondin 3	-0.89	0.04
SEMA3F	semaphorin 3F	-0.88	0.03
NLRC5	NLR family CARD domain containing 5	-0.88	0.02
MAP1B	microtubule associated protein 1B	-0.88	0.00
NSG1	neuronal vesicle trafficking associated 1	-0.88	0.05
RAC2	Rac family small GTPase 2	-0.88	0.04
APAF1	apoptotic peptidase activating factor 1	-0.88	0.02
POC5	POC5 centriolar protein	-0.87	0.03
SHANK3	SH3 and multiple ankyrin repeat domains 3	-0.86	0.02
gene:ENSSSCG00000007435		-0.85	0.05
CHRD	chordin	-0.84	0.02

SERTM1	serine rich and transmembrane domain containing 1	-0.83	0.02
ASS1	argininosuccinate synthase 1	-0.83	0.01
HIVEP2	human immunodeficiency virus type I enhancer binding protein 2	-0.82	0.00
ZHX2	zinc fingers and homeoboxes 2	-0.82	0.01
ZBTB40	zinc finger and BTB domain containing 40	-0.82	0.03
MAST4	microtubule associated serine/threonine kinase family member 4	-0.81	0.01
CORO2B	coronin 2B	-0.80	0.02
MX2	MX dynamin like GTPase 2	-0.80	0.01
TLNRD1	talin rod domain containing 1	-0.79	0.01
CORO1A	coronin 1A	-0.79	0.02
MPP3	membrane palmitoylated protein 3	-0.79	0.00
GXYLT1	glucoside xylosyltransferase 1	-0.78	0.03
L1CAM	L1 cell adhesion molecule	-0.78	0.03
RNF213	ring finger protein 213	-0.78	0.03
TNS2	tensin 2	-0.77	0.01
PANK1	pantothenate kinase 1	-0.77	0.02
gene:ENSSSCG00000001396		-0.77	0.02
IFIT2	interferon induced protein with tetratricopeptide repeats 2	-0.76	0.01
EHBP1L1	EH domain binding protein 1 like 1	-0.76	0.02
AGRN	agrin	-0.76	0.03
TYK2	tyrosine kinase 2	-0.76	0.00
gene:ENSSSCG00000040337		-0.76	0.03
SYVN1	synoviolin 1	-0.76	0.01
PFKP	phosphofructokinase, platelet	-0.75	0.00
F8	coagulation factor VIII	-0.75	0.00
GTSE1	G2 and S-phase expressed 1	-0.74	0.03
SPARC	secreted protein acidic and cysteine rich	-0.74	0.05
PLEKHG5	pleckstrin homology and RhoGEF domain containing G5	-0.74	0.03
MAP3K12	mitogen-activated protein kinase kinase kinase 12	-0.73	0.05

TSPO	translocator protein	-0.73	0.03
NADSYN1	NAD synthetase 1	-0.73	0.02
RECK	reversion inducing cysteine rich protein with kazal motifs	-0.72	0.04
CTSW	cathepsin W	-0.72	0.03
gene:ENSSSCG00000038260		-0.72	0.03
HERC6	HECT and RLD domain containing E3 ubiquitin protein ligase family member 6	-0.71	0.04
gene:ENSSSCG00000009293		-0.70	0.03
TNFRSF19	TNF receptor superfamily member 19	-0.70	0.03
COL5A1	collagen type V alpha 1 chain	-0.69	0.04
ITGB2	integrin subunit beta 2	-0.68	0.05
COL5A2	collagen type V alpha 2 chain	-0.68	0.04
PHRF1	PHD and ring finger domains 1	-0.68	0.03
PCDHGA9	protocadherin gamma subfamily A, 9	-0.68	0.04
EBF3	early B cell factor 3	-0.68	0.03
TCIRG1	T cell immune regulator 1, ATPase H+ transporting V0 subunit a3	-0.68	0.03
SLC5A6	solute carrier family 5 member 6	-0.68	0.01
B3GLCT	beta 3-glucosyltransferase	-0.68	0.04
SRRM2	serine/arginine repetitive matrix 2	-0.67	0.00
NOS3	nitric oxide synthase 3	-0.67	0.04
PLAT	plasminogen activator, tissue type	-0.67	0.05
IGFBP4	insulin like growth factor binding protein 4	-0.67	0.05
MTMR4	myotubularin related protein 4	-0.66	0.01
ABCC10	ATP binding cassette subfamily C member 10	-0.66	0.01
SBNO2	strawberry notch homolog 2	-0.66	0.03
DPH2	DPH2 homolog	-0.66	0.04
RAP1GDS1	Rap1 GTPase-GDP dissociation stimulator 1	-0.66	0.03
ING5	inhibitor of growth family member 5	-0.66	0.02
FHOD1	formin homology 2 domain containing 1	-0.66	0.03
KAT7	lysine acetyltransferase 7	-0.66	0.03
IL4R	interleukin 4 receptor	-0.65	0.02

EPB41L4B	erythrocyte membrane protein band 4.1 like 4B	-0.65	0.04
ADCK5	aarF domain containing kinase 5	-0.65	0.04
ABLIM2	actin binding LIM protein family member 2	-0.65	0.01
PEAR1	platelet endothelial aggregation receptor 1	-0.65	0.02
STARD9	StAR related lipid transfer domain containing 9	-0.64	0.03
gene:ENSSSCG00000037530		-0.64	0.03
KAT6B	lysine acetyltransferase 6B	-0.64	0.02
CHD7	chromodomain helicase DNA binding protein 7	-0.63	0.02
JAG1	jagged 1	-0.63	0.02
RAPGEF5	Rap guanine nucleotide exchange factor 5	-0.63	0.02
ZC3H18	zinc finger CCCH-type containing 18	-0.62	0.04
LAMA5	laminin subunit alpha 5	-0.62	0.04
UNK	unkempt family zinc finger	-0.62	0.04
BRPF3	bromodomain and PHD finger containing 3	-0.61	0.03
CIC	capicua transcriptional repressor	-0.61	0.02
HRH1	histamine receptor H1	-0.61	0.02
CCDC6	coiled-coil domain containing 6	-0.61	0.04
DLG5	discs large MAGUK scaffold protein 5	-0.61	0.04
CREBBP	CREB binding protein	-0.61	0.04
HAUS5	HAUS augmin like complex subunit 5	-0.60	0.02
B3GNT3	UDP-GlcNAc:betaGal beta-1,3-N-acetylglucosaminyltransferase 3	-0.60	0.02
PGD	phosphogluconate dehydrogenase	-0.60	0.03
TMEM2	transmembrane protein 2	-0.60	0.05
CARD10	caspase recruitment domain family member 10	-0.60	0.04
EP300	E1A binding protein p300	-0.60	0.01
gene:ENSSSCG00000028278		-0.59	0.03

HSPG2	heparan sulfate proteoglycan 2	-0.59	0.01
MCF2L	MCF.2 cell line derived transforming sequence like	-0.59	0.03
ATG2A	autophagy related 2A	-0.59	0.02
ZFP36L1	ZFP36 ring finger protein like 1	-0.59	0.03

Supplemental Table S3.

Selected signature Sus Scrofa gene expressions altered > 1.5-fold change (Log_2FC (RVOT-VPC/sham control) > 0.58) in the left ventricular free wall tissues of RVOT VPC group vs. sham control group at 6-month follow-up

Symbol	Description	Log_2 FC	P value
gene:ENSSSCG00000036324		5.81	0.02
UBXN10	UBX domain protein 10	5.66	0.02
TSNAXIP1	translin associated factor X interacting protein 1	5.16	0.04
gene:ENSSSCG00000038101		4.94	0.04
SYT1	synaptotagmin 1	4.39	0.00
SCN3B	sodium voltage-gated channel beta subunit 3	3.81	0.00
OAZ3	ornithine decarboxylase antizyme 3	3.80	0.00
PDYN	prodynorphin	3.70	0.00
WWC1	WW and C2 domain containing 1	3.65	0.00
UMODL1	uromodulin like 1	3.44	0.00
GDNF	glial cell derived neurotrophic factor	3.34	0.00
MDGA2	MAM domain containing glycosylphosphatidylinositol anchor 2	3.29	0.04
gene:ENSSSCG00000035226		3.28	0.03
SH3GL3	SH3 domain containing GRB2 like 3, endophilin A3	3.27	0.02
CTNND2	catenin delta 2	3.08	0.00
SYT5	synaptotagmin 5	3.05	0.03
gene:ENSSSCG00000008205		2.93	0.01
gene:ENSSSCG00000017463		2.80	0.04
IRX1	iroquois homeobox 1	2.76	0.02
SYPL2	synaptophysin like 2	2.67	0.00
CPNE6	copine 6	2.67	0.02
gene:ENSSSCG00000030013		2.67	0.02

PTGS2	prostaglandin-endoperoxide synthase 2	2.64	0.00
VSIG2	V-set and immunoglobulin domain containing 2	2.62	0.00
FOSB	FosB proto-oncogene, AP-1 transcription factor subunit	2.62	0.00
SLN	sarcolipin	2.61	0.00
ARC	activity regulated cytoskeleton associated protein	2.59	0.01
gene:ENSSSCG00000000021		2.58	0.02
SLC18A1	solute carrier family 18 member A1	2.58	0.02
gene:ENSSSCG00000038690		2.57	0.03
gene:ENSSSCG00000040198		2.55	0.03
IRX2	iroquois homeobox 2	2.50	0.00
KCNG2	potassium voltage-gated channel modifier subfamily G member 2	2.48	0.03
RGS6	regulator of G protein signaling 6	2.47	0.00
RIMBP2	RIMS binding protein 2	2.43	0.00
gene:ENSSSCG00000036109		2.43	0.02
CCDC190	coiled-coil domain containing 190	2.40	0.02
gene:ENSSSCG00000021271		2.38	0.00
IRX6	iroquois homeobox 6	2.37	0.00
gene:ENSSSCG00000010764		2.32	0.02
GPT2	glutamic--pyruvic transaminase 2	2.31	0.02
UNC13A	unc-13 homolog A	2.30	0.00
gene:ENSSSCG00000008965		2.29	0.04
EGR1	early growth response 1	2.28	0.00
UGT8	UDP glycosyltransferase 8	2.26	0.05
TRIM46	tripartite motif containing 46	2.26	0.04
ATCAY	ATCAY, caytaxin	2.26	0.05
gene:ENSSSCG00000029866		2.26	0.00

gene:ENSSSCG00000001550		2.25	0.03
SLC28A1	solute carrier family 28 member 1	2.24	0.01
CCL2	C-C motif chemokine ligand 2	2.24	0.00
NNAT	neuronatin	2.24	0.00
IL1RL2	interleukin 1 receptor like 2	2.21	0.03
DKK3	dickkopf WNT signaling pathway inhibitor 3	2.20	0.00
NPPB	natriuretic peptide B	2.20	0.00
CENPS	centromere protein S	2.18	0.01
ATF3	activating transcription factor 3	2.16	0.00
GPR156	G protein-coupled receptor 156	2.16	0.03
PADI2	peptidyl arginine deiminase 2	2.15	0.00
IL13RA2	interleukin 13 receptor subunit alpha 2	2.14	0.02
DUSP5	dual specificity phosphatase 5	2.13	0.00
CSGALNACT1	chondroitin sulfate N-acetylgalactosaminyltransferase 1	2.10	0.01
IL23A	interleukin 23 subunit alpha	2.10	0.04
OSR2	odd-skipped related transciption factor 2	2.08	0.00
CD209	CD209 molecule	2.07	0.00
GRAMD1B	GRAM domain containing 1B	2.07	0.00
UBE2C	ubiquitin conjugating enzyme E2 C	2.07	0.05
CXCL2	C-X-C motif chemokine ligand 2	2.07	0.00
gene:ENSSSCG00000040939		2.06	0.02
MYL4	myosin light chain 4	2.06	0.00
gene:ENSSSCG00000031997		2.05	0.04
NIPAL4	NIPA like domain containing 4	2.03	0.02
gene:ENSSSCG00000039066		2.01	0.04
LRRN1	leucine rich repeat neuronal 1	2.00	0.02
gene:ENSSSCG00000023403		2.00	0.01
GDF10	growth differentiation factor 10	1.99	0.02
CDCA7	cell division cycle associated 7	1.99	0.02
CDH12	cadherin 12	1.98	0.01

SOCS3	suppressor of cytokine signaling 3	1.97	0.00
ARG2	arginase 2	1.95	0.00
gene:ENSSSCG00000040453		1.95	0.00
KEL	Kell blood group, metallo-endopeptidase	1.94	0.03
SLC9A2	solute carrier family 9 member A2	1.93	0.00
TRPV5	transient receptor potential cation channel subfamily V member 5	1.93	0.03
TNNI1	troponin I1, slow skeletal type	1.92	0.01
gene:ENSSSCG00000039214		1.87	0.04
NPPA	natriuretic peptide A	1.85	0.00
gene:ENSSSCG00000011324		1.85	0.00
SPDEF	SAM pointed domain containing ETS transcription factor	1.84	0.01
SLC15A2	solute carrier family 15 member 2	1.83	0.02
STRC	stereocilin	1.82	0.00
ROR1	receptor tyrosine kinase like orphan receptor 1	1.82	0.00
HBM	hemoglobin subunit mu	1.82	0.05
SMCO1	single-pass membrane protein with coiled-coil domains 1	1.81	0.01
gene:ENSSSCG00000014272		1.81	0.00
gene:ENSSSCG00000038357		1.81	0.01
MNS1	meiosis specific nuclear structural 1	1.81	0.00
IFN-OMEGA-7		1.79	0.05
DUOX2	dual oxidase 2	1.79	0.03
NRSN1	neurensin 1	1.78	0.00
RAB7B	RAB7B, member RAS oncogene family	1.78	0.00
IL10RA	interleukin 10 receptor subunit alpha	1.77	0.00
MALRD1	MAM and LDL receptor class A domain containing 1	1.75	0.01
GADL1	glutamate decarboxylase like 1	1.74	0.00

gene:ENSSSCG00000034761		1.73	0.02
GRIK4	glutamate ionotropic receptor kainate type subunit 4	1.70	0.01
MYMK	myomaker, myoblast fusion factor	1.69	0.00
RAB6B	RAB6B, member RAS oncogene family	1.68	0.01
gene:ENSSSCG00000034812		1.66	0.01
P2RX1	purinergic receptor P2X 1	1.64	0.02
ADRA1D	adrenoceptor alpha 1D	1.64	0.03
BEX4	brain expressed X-linked 4	1.63	0.02
SAYSD1	SAYSVFN motif domain containing 1	1.63	0.00
CCDC173	coiled-coil domain containing 173	1.60	0.00
gene:ENSSSCG00000009839		1.58	0.03
SLC39A12	solute carrier family 39 member 12	1.58	0.04
CROCC2	ciliary rootlet coiled-coil, rootletin family member 2	1.58	0.01
SPATA18	spermatogenesis associated 18	1.56	0.05
NR4A1	nuclear receptor subfamily 4 group A member 1	1.54	0.00
RF02159		1.52	0.01
MAATS1	MYCBP associated and testis expressed 1	1.51	0.04
gene:ENSSSCG00000009500		1.50	0.01
FOS	Fos proto-oncogene, AP-1 transcription factor subunit	1.50	0.03
ACSM3	acyl-CoA synthetase medium chain family member 3	1.49	0.02
gene:ENSSSCG00000039704		1.48	0.01
CDH16	cadherin 16	1.47	0.03
VANGL2	VANGL planar cell polarity protein 2	1.47	0.02
gene:ENSSSCG00000018015		1.46	0.03
gene:ENSSSCG0000000895		1.45	0.01

4			
KLRG2	killer cell lectin like receptor G2	1.45	0.03
LRRC4B	leucine rich repeat containing 4B	1.45	0.00
SYT17	synaptotagmin 17	1.44	0.01
PHACTR3	phosphatase and actin regulator 3	1.42	0.00
CTGF	connective tissue growth factor	1.42	0.00
SYT13	synaptotagmin 13	1.42	0.03
L3HYPDH	trans-L-3-hydroxyproline dehydratase	1.41	0.00
EXD1	exonuclease 3'-5' domain containing 1	1.41	0.01
FGF16	fibroblast growth factor 16	1.40	0.03
TACR1	tachykinin receptor 1	1.39	0.02
FGFRL1	fibroblast growth factor receptor like 1	1.38	0.00
COL13A1	collagen type XIII alpha 1 chain	1.38	0.02
GDF11	growth differentiation factor 11	1.37	0.02
FRAS1	Fraser extracellular matrix complex subunit 1	1.37	0.00
MID1	midline 1	1.37	0.02
ARNT2	aryl hydrocarbon receptor nuclear translocator 2	1.37	0.01
EYA1	EYA transcriptional coactivator and phosphatase 1	1.36	0.02
MYOZ1	myozenin 1	1.36	0.04
FMO5	flavin containing monooxygenase 5	1.36	0.02
ITGAE	integrin subunit alpha E	1.36	0.00
SHAS2		1.35	0.01
TANC2	tetratricopeptide repeat, ankyrin repeat and coiled-coil containing 2	1.35	0.01
PHLDA1	pleckstrin homology like domain family A member 1	1.33	0.01
EGR2	early growth response 2	1.33	0.00
PLEK2	pleckstrin 2	1.32	0.02
EDIL3	EGF like repeats and discoidin domains 3	1.32	0.00
IL6	interleukin 6	1.32	0.03
RAB17	RAB17, member RAS oncogene family	1.30	0.03
FRMD1	FERM domain containing 1	1.30	0.02
ADAMTS14	ADAM metallopeptidase with	1.30	0.01

	thrombospondin type 1 motif 14		
GPNMB	glycoprotein nmb	1.28	0.01
GPC4	glypican 4	1.28	0.00
MILR1	mast cell immunoglobulin like receptor 1	1.28	0.05
TNFAIP6	TNF alpha induced protein 6	1.28	0.01
ARG1	arginase 1	1.28	0.05
FGD6	FYVE, RhoGEF and PH domain containing 6	1.27	0.00
PTN	pleiotrophin	1.27	0.04
gene:ENSSSCG00000032594		1.27	0.00
SIGLEC1	sialic acid binding Ig like lectin 1	1.26	0.04
PIWIL4	piwi like RNA-mediated gene silencing 4	1.26	0.03
CSRNP1	cysteine and serine rich nuclear protein 1	1.25	0.00
SLC6A4	solute carrier family 6 member 4	1.23	0.01
EFCC1	EF-hand and coiled-coil domain containing 1	1.23	0.02
LOXL4	lysyl oxidase like 4	1.22	0.02
PCSK4	proprotein convertase subtilisin/kexin type 4	1.22	0.02
ATP4A	ATPase H+/K+ transporting alpha subunit	1.22	0.05
MBOAT2	membrane bound O-acyltransferase domain containing 2	1.21	0.00
CHST1	carbohydrate sulfotransferase 1	1.19	0.01
NR4A3	nuclear receptor subfamily 4 group A member 3	1.17	0.00
gene:ENSSSCG00000034266		1.17	0.01
XKRX	XK related, X-linked	1.17	0.04
IER5	immediate early response 5	1.17	0.00
gene:ENSSSCG00000040693		1.17	0.02
INHA	inhibin alpha subunit	1.16	0.00
NEU3	neuraminidase 3	1.16	0.00

EHD3	EH domain containing 3	1.15	0.00
UCHL1	ubiquitin C-terminal hydrolase L1	1.15	0.01
TMCO4	transmembrane and coiled-coil domains 4	1.14	0.05
HMGCLL1	3-hydroxymethyl-3-methylglutaryl-CoA lyase like 1	1.14	0.04
CDKL3	cyclin dependent kinase like 3	1.14	0.02
PSD	pleckstrin and Sec7 domain containing	1.13	0.05
C2orf76	chromosome 2 open reading frame 76	1.12	0.03
gene:ENSSSCG00000010278		1.12	0.00
gene:ENSSSCG00000032301		1.12	0.04
TCTN3	tectonic family member 3	1.10	0.02
PHEX	phosphate regulating endopeptidase homolog X-linked	1.07	0.01
EPB41L5	erythrocyte membrane protein band 4.1 like 5	1.07	0.00
FBP1	fructose-bisphosphatase 1	1.06	0.02
NAAA	N-acylethanolamine acid amidase	1.06	0.00
VWA5A	von Willebrand factor A domain containing 5A	1.06	0.00
IRX5	iroquois homeobox 5	1.06	0.03
ZSWIM4	zinc finger SWIM-type containing 4	1.05	0.01
LGALS3	galectin 3	1.05	0.01
TP63	tumor protein p63	1.05	0.01
CYFIP2	cytoplasmic FMR1 interacting protein 2	1.05	0.00
CDKL1	cyclin dependent kinase like 1	1.05	0.00
ETV1	ETS variant 1	1.04	0.00
PLAUR	plasminogen activator, urokinase receptor	1.04	0.02
FRZB	frizzled related protein	1.03	0.03
PLIN5	perilipin 5	1.03	0.02
MASP1	mannan binding lectin serine peptidase 1	1.02	0.00
BCAT1	branched chain amino acid transaminase 1	1.01	0.01

ATP13A3	ATPase 13A3	1.01	0.00
HABP2	hyaluronan binding protein 2	1.00	0.05
FBLN5	fibulin 5	0.99	0.02
GNB3	G protein subunit beta 3	0.99	0.00
RIPOR2	RHO family interacting cell polarization regulator 2	0.99	0.00
ADGRF2	adhesion G protein-coupled receptor F2	0.98	0.05
C12orf75	chromosome 12 open reading frame 75	0.98	0.01
CHMP4C	charged multivesicular body protein 4C	0.97	0.01
FMO2	flavin containing monooxygenase 2	0.96	0.03
SERPINF1	serpin family F member 1	0.96	0.00
FAM46B	family with sequence similarity 46 member B	0.95	0.02
gene:ENSSSCG00000015293		0.95	0.04
BTG2	BTG anti-proliferation factor 2	0.95	0.05
TGIF1	TGFB induced factor homeobox 1	0.94	0.01
DNER	delta/notch like EGF repeat containing	0.94	0.05
IGFBP3	insulin like growth factor binding protein 3	0.93	0.03
DUSP2	dual specificity phosphatase 2	0.93	0.03
ITGA11	integrin subunit alpha 11	0.92	0.01
MAFF	MAF bZIP transcription factor F	0.92	0.01
KRT80	keratin 80	0.91	0.02
gene:ENSSSCG00000004388		0.91	0.03
EPGN	epithelial mitogen	0.91	0.05
C4BPA	complement component 4 binding protein alpha	0.90	0.00
RF02160		0.90	0.04
NR4A2	nuclear receptor subfamily 4 group A member 2	0.90	0.01
BCL6	B cell CLL/lymphoma 6	0.90	0.00
FEM1C	fem-1 homolog C	0.89	0.00
gene:ENSSSCG00000031450		0.89	0.00

KIAA1324L	KIAA1324 like	0.88	0.00
PHACTR1	phosphatase and actin regulator 1	0.88	0.02
RGS1	regulator of G protein signaling 1	0.88	0.02
PLXDC1	plexin domain containing 1	0.87	0.01
AOX1	aldehyde oxidase 1	0.87	0.03
CDON	cell adhesion associated, oncogene regulated	0.87	0.00
IER3	immediate early response 3	0.87	0.03
ADCY5	adenylate cyclase 5	0.87	0.00
gene:ENSSSCG00000003451		0.87	0.03
SGSM1	small G protein signaling modulator 1	0.86	0.04
STX11	syntaxin 11	0.86	0.05
CX3CL1	C-X3-C motif chemokine ligand 1	0.86	0.02
CARD11	caspase recruitment domain family member 11	0.86	0.02
KLF6	Kruppel like factor 6	0.86	0.03
MEST	mesoderm specific transcript	0.86	0.05
CTSC	cathepsin C	0.86	0.00
SH3RF1	SH3 domain containing ring finger 1	0.86	0.05
KLHL32	kelch like family member 32	0.85	0.01
IGSF8	immunoglobulin superfamily member 8	0.85	0.02
RNF180	ring finger protein 180	0.85	0.00
gene:ENSSSCG00000011041		0.85	0.02
SERPINE1	serpin family E member 1	0.84	0.01
CCND2	cyclin D2	0.83	0.01
GCNT2	glucosaminyl (N-acetyl) transferase 2, I-branching enzyme (I blood group)	0.82	0.00
SDR39U1	short chain dehydrogenase/reductase family 39U member 1	0.81	0.01
KLF10	Kruppel like factor 10	0.81	0.00
WSCD2	WSC domain containing 2	0.81	0.02
gene:ENSSSCG00000023611		0.80	0.00
ADRB2	adrenoceptor beta 2	0.80	0.03
PPP2R1B	protein phosphatase 2 scaffold subunit	0.79	0.01

	Abeta		
SLC51B	solute carrier family 51 beta subunit	0.79	0.05
DEPP1	DEPP1, autophagy regulator	0.79	0.00
MGST1	microsomal glutathione S-transferase 1	0.79	0.00
OLFM1	olfactomedin 1	0.78	0.04
CYB5A	cytochrome b5 type A	0.78	0.00
TNIK	TRAF2 and NCK interacting kinase	0.78	0.00
KCNA5	potassium voltage-gated channel subfamily A member 5	0.78	0.02
HTRA3	HtrA serine peptidase 3	0.77	0.01
RTN4RL1	reticulon 4 receptor like 1	0.77	0.00
SFRP1	secreted frizzled related protein 1	0.77	0.00
gene:ENSSSCG00000003253		0.77	0.00
CCDC18	coiled-coil domain containing 18	0.76	0.04
VSIG4	V-set and immunoglobulin domain containing 4	0.76	0.01
WFS1	wolframin ER transmembrane glycoprotein	0.75	0.00
HIST1H2BD	histone cluster 1 H2B family member d	0.75	0.00
IFRD1	interferon related developmental regulator 1	0.75	0.00
FKBP10	FK506 binding protein 10	0.74	0.03
DCBLD2	discoidin, CUB and LCCL domain containing 2	0.73	0.03
RCAN1	regulator of calcineurin 1	0.72	0.03
CASQ1	calsequestrin 1	0.72	0.02
CREM	cAMP responsive element modulator	0.72	0.02
gene:ENSSSCG00000008440		0.71	0.01
HEBP1	heme binding protein 1	0.71	0.01
NABP1	nucleic acid binding protein 1	0.71	0.02
CCDC136	coiled-coil domain containing 136	0.71	0.04
LAP3	leucine aminopeptidase 3	0.71	0.00
COL4A5	collagen type IV alpha 5 chain	0.70	0.00
F3	coagulation factor III, tissue factor	0.70	0.01
KLF4	Kruppel like factor 4	0.69	0.00

SERPINE2	serpin family E member 2	0.69	0.00
IRF2BP2	interferon regulatory factor 2 binding protein 2	0.69	0.00
FGF13	fibroblast growth factor 13	0.68	0.00
NDST3	N-deacetylase and N-sulfotransferase 3	0.68	0.04
ZFP36	ZFP36 ring finger protein	0.67	0.03
GRAMD1C	GRAM domain containing 1C	0.67	0.04
SAT1	spermidine/spermine N1-acetyltransferase 1	0.66	0.00
ALDH5A1	aldehyde dehydrogenase 5 family member A1	0.66	0.00
ARHGAP23	Rho GTPase activating protein 23	0.66	0.02
PDGFD	platelet derived growth factor D	0.66	0.01
GPC3	glypican 3	0.65	0.02
FABP3	fatty acid binding protein 3	0.64	0.00
GGACT	gamma-glutamylamine cyclotransferase	0.63	0.03
SLC29A1	solute carrier family 29 member 1 (Augustine blood group)	0.63	0.00
FEZ2	fasciculation and elongation protein zeta 2	0.63	0.01
FBLIM1	filamin binding LIM protein 1	0.62	0.04
FOLR1	folate receptor 1	0.62	0.01
C1QA	complement C1q A chain	0.62	0.04
PLXNA4	plexin A4	0.62	0.03
BHLHE40	basic helix-loop-helix family member e40	0.62	0.03
FOSL2	FOS like 2, AP-1 transcription factor subunit	0.61	0.01
TPM2	tropomyosin 2	0.61	0.00
STXBP6	syntaxin binding protein 6	0.61	0.03
RTL5	retrotransposon Gag like 5	0.61	0.01
SLC38A2	solute carrier family 38 member 2	0.61	0.01
TCF21	transcription factor 21	0.60	0.02
NDEL1	nudE neurodevelopment protein 1 like 1	0.60	0.01
CASZ1	castor zinc finger 1	0.60	0.03
PRKDC	protein kinase, DNA-activated,	0.60	0.03

	catalytic polypeptide		
TPPP3	tubulin polymerization promoting protein family member 3	0.59	0.02
IL6R	interleukin 6 receptor	0.59	0.00
KDELC1	KDEL motif containing 1	0.59	0.04

Supplemental Table S4.

Selected signature Sus Scrofa gene expressions altered < 0.66-fold change (Log_2FC (RVOT-VPC/sham control) < −0.58) in the left ventricular free wall tissues of RVOT VPC group vs. sham control group at 6-month follow-up

Symbol	Description	Log_2 FC	P value
LIPM	lipase family member M	-7.97	0.00
RLBP1	retinaldehyde binding protein 1	-7.30	0.00
gene:ENSSSCG00000034022		-5.71	0.03
RIMS3	regulating synaptic membrane exocytosis 3	-5.60	0.02
gene:ENSSSCG00000020695		-5.21	0.00
MMP9	matrix metallopeptidase 9	-5.14	0.04
GALR3	galanin receptor 3	-5.00	0.04
CLSTN2	calsyntenin 2	-4.72	0.05
FADS6	fatty acid desaturase 6	-4.54	0.00
WIF1	WNT inhibitory factor 1	-4.46	0.00
CDHR1	cadherin related family member 1	-4.41	0.00
GPRL15		-3.91	0.01
CRLF1	cytokine receptor like factor 1	-3.77	0.00
RF00004_25		-3.67	0.01
gene:ENSSSCG00000035996		-3.59	0.00
MMP8	matrix metallopeptidase 8	-3.56	0.02
gene:ENSSSCG00000032652		-3.54	0.02
LCAT	lecithin-cholesterol acyltransferase	-3.49	0.02
gene:ENSSSCG00000034356		-3.44	0.00
gene:ENSSSCG00000034253		-3.41	0.00
gene:ENSSSCG00000016475		-3.40	0.00
HOXB7	homeobox B7	-3.35	0.01
SYCE1	synaptonemal complex central element protein 1	-3.31	0.04
CACNA1S	calcium voltage-gated channel subunit alpha1 S	-3.30	0.00
NMB	neuromedin B	-3.28	0.02
ANGPTL7	angiopoietin like 7	-3.22	0.00
S100A8	S100 calcium binding protein A8	-3.18	0.00
gene:ENSSSCG00000026402		-3.16	0.03
IFN-ALPHA-16		-3.14	0.03

TMIGD1	transmembrane and immunoglobulin domain containing 1	-3.09	0.01
gene:ENSSSCG00000029705		-3.02	0.03
gene:ENSSSCG00000016245		-2.98	0.01
LTF	lactotransferrin	-2.92	0.00
S100A12	S100 calcium binding protein A12	-2.78	0.00
NAT8L	N-acetyltransferase 8 like	-2.78	0.01
MAP3K9	mitogen-activated protein kinase kinase kinase 9	-2.71	0.05
PCLAF	PCNA clamp associated factor	-2.71	0.02
KRT18	keratin 18	-2.71	0.04
ACTG2	actin, gamma 2, smooth muscle, enteric	-2.59	0.00
PLIN1	perilipin 1	-2.59	0.03
TNC	tenascin C	-2.48	0.00
GPR37	G protein-coupled receptor 37	-2.48	0.02
NALCN	sodium leak channel, non-selective	-2.46	0.00
gene:ENSSSCG00000001231		-2.45	0.00
gene:ENSSSCG00000032596		-2.43	0.03
S100A9	S100 calcium binding protein A9	-2.41	0.00
gene:ENSSSCG00000013788		-2.34	0.00
gene:ENSSSCG00000038068		-2.34	0.00
gene:ENSSSCG00000031292		-2.34	0.03
KCNMB1	potassium calcium-activated channel subfamily M regulatory beta subunit 1	-2.31	0.00
CA3	carbonic anhydrase 3	-2.29	0.01
gene:ENSSSCG00000006382		-2.28	0.00
STAC2	SH3 and cysteine rich domain 2	-2.27	0.01
CYP3A39		-2.20	0.00
PKP1	plakophilin 1	-2.11	0.04
ANKRD22	ankyrin repeat domain 22	-2.11	0.01
gene:ENSSSCG00000038557		-2.11	0.00
ARHGEF16	Rho guanine nucleotide exchange factor 16	-2.10	0.04
DMRTA1	DMRT like family A1	-2.09	0.04
KRT14	keratin 14	-2.08	0.05
SLC9A3	solute carrier family 9 member A3	-2.08	0.03

HOXA5	homeobox A5	-2.05	0.01
FASLG	Fas ligand	-2.04	0.02
ITGBL1	integrin subunit beta like 1	-2.02	0.00
XCL1	X-C motif chemokine ligand 1	-2.02	0.03
MT3	metallothionein 3	-2.01	0.00
gene:ENSSSCG00000029414		-2.00	0.00
CD3D	CD3d molecule	-1.99	0.00
GZMB	granzyme B	-1.96	0.00
gene:ENSSSCG00000013434		-1.95	0.00
gene:ENSSSCG00000023746		-1.95	0.05
gene:ENSSSCG00000031902		-1.94	0.01
EMB	embigin	-1.93	0.05
IFN-ALPHA-8		-1.93	0.04
TBC1D10C	TBC1 domain family member 10C	-1.92	0.01
CD6	CD6 molecule	-1.91	0.00
gene:ENSSSCG00000031610		-1.91	0.01
SYTL1	synaptotagmin like 1	-1.84	0.04
KCNJ5	potassium voltage-gated channel subfamily J member 5	-1.83	0.00
SLC1A1	solute carrier family 1 member 1	-1.83	0.00
PVRIG	PVR related immunoglobulin domain containing	-1.82	0.05
MT1A	metallothionein 1A	-1.81	0.01
IL7R	interleukin 7 receptor	-1.81	0.03
TREML2	triggering receptor expressed on myeloid cells like 2	-1.81	0.04
DDAH1	dimethylarginine dimethylaminohydrolase 1	-1.79	0.00
gene:ENSSSCG00000017018		-1.79	0.00
DAPP1	dual adaptor of phosphotyrosine and 3-phosphoinositides 1	-1.79	0.03
CST7	cystatin F	-1.78	0.02
gene:ENSSSCG00000037676		-1.78	0.00
CD96	CD96 molecule	-1.76	0.04
SEMA3A	semaphorin 3A	-1.74	0.05
IFNLR1	interferon lambda receptor 1	-1.73	0.03
CDCA7L	cell division cycle associated 7 like	-1.72	0.00
DNAJC6	DnaJ heat shock protein family	-1.67	0.00

	(Hsp40) member C6		
FOXC1	forkhead box C1	-1.67	0.02
SLC6A17	solute carrier family 6 member 17	-1.67	0.01
CCL19	C-C motif chemokine ligand 19	-1.67	0.01
MSTN	myostatin	-1.67	0.01
gene:ENSSSCG00000040295		-1.65	0.03
MMRN2	multimerin 2	-1.62	0.00
PRF1	perforin 1	-1.61	0.01
MYL1	myosin light chain 1	-1.61	0.01
KCNC4	potassium voltage-gated channel subfamily C member 4	-1.60	0.02
CD5	CD5 molecule	-1.59	0.01
CD69	CD69 molecule	-1.59	0.00
ICOS	inducible T cell costimulator	-1.59	0.05
CD52	CD52 molecule	-1.58	0.00
CYP2S1	cytochrome P450 family 2 subfamily S member 1	-1.56	0.03
CX3CR1	C-X3-C motif chemokine receptor 1	-1.56	0.00
gene:ENSSSCG00000033721		-1.55	0.01
GALNT13	polypeptide N-acetylgalactosaminyltransferase 13	-1.55	0.03
SFRP5	secreted frizzled related protein 5	-1.53	0.01
KANK4	KN motif and ankyrin repeat domains 4	-1.53	0.04
SERTAD4	SERTA domain containing 4	-1.53	0.01
MYO1G	myosin IG	-1.51	0.01
GYG2	glycogenin 2	-1.50	0.03
SNX22	sorting nexin 22	-1.50	0.00
gene:ENSSSCG00000039222		-1.50	0.04
LPXN	leupaxin	-1.49	0.03
gene:ENSSSCG00000037885		-1.49	0.01
SIT1	signaling threshold regulating transmembrane adaptor 1	-1.47	0.03
gene:ENSSSCG00000040410		-1.46	0.04
gene:ENSSSCG00000020967		-1.45	0.02
SCG3	secretogranin III	-1.43	0.02
CD2	CD2 molecule	-1.41	0.00

SALL4	spalt like transcription factor 4	-1.40	0.03
gene:ENSSSCG00000035256		-1.36	0.00
gene:ENSSSCG00000002140		-1.34	0.00
ANPEP	alanyl aminopeptidase, membrane	-1.32	0.05
PYGO1	pygopus family PHD finger 1	-1.31	0.02
MYH11	myosin heavy chain 11	-1.30	0.04
gene:ENSSSCG00000028341		-1.29	0.02
CCL5	C-C motif chemokine ligand 5	-1.29	0.01
IKZF3	IKAROS family zinc finger 3	-1.27	0.01
gene:ENSSSCG00000040350		-1.26	0.02
KIF21B	kinesin family member 21B	-1.26	0.01
RASAL3	RAS protein activator like 3	-1.26	0.04
GNLY	granulysin	-1.26	0.00
CD3E	CD3e molecule	-1.25	0.03
TMEM102	transmembrane protein 102	-1.25	0.04
TRIM25	tripartite motif containing 25	-1.22	0.01
APOE	apolipoprotein E	-1.21	0.02
DKK1	dickkopf WNT signaling pathway inhibitor 1	-1.19	0.03
SKAP1	src kinase associated phosphoprotein 1	-1.19	0.03
FAM43B	family with sequence similarity 43 member B	-1.18	0.05
ASS1	argininosuccinate synthase 1	-1.17	0.00
GIMAP8	GTPase, IMAP family member 8	-1.17	0.02
ROBO3	roundabout guidance receptor 3	-1.16	0.04
CPXM1	carboxypeptidase X, M14 family member 1	-1.15	0.01
CDH24	cadherin 24	-1.14	0.01
KIF18B	kinesin family member 18B	-1.14	0.04
MMP28	matrix metallopeptidase 28	-1.14	0.02
IFIT2	interferon induced protein with tetratricopeptide repeats 2	-1.14	0.00
gene:ENSSSCG00000017019		-1.13	0.04
SRARP	steroid receptor associated and regulated protein	-1.13	0.02
GCAT	glycine C-acetyltransferase	-1.12	0.01
gene:ENSSSCG00000008898		-1.12	0.00

UBASH3B	ubiquitin associated and SH3 domain containing B	-1.11	0.00
SEMA4A	semaphorin 4A	-1.11	0.03
CD8A	CD8a molecule	-1.10	0.02
INMT	indolethylamine N-methyltransferase	-1.09	0.02
SLA2	Src like adaptor 2	-1.09	0.02
gene:ENSSSCG00000029596		-1.08	0.02
INPP1	inositol polyphosphate-1-phosphatase	-1.06	0.00
SMPD3	sphingomyelin phosphodiesterase 3	-1.05	0.05
POC5	POC5 centriolar protein	-1.05	0.00
gene:ENSSSCG00000012531		-1.05	0.03
AEBP1	AE binding protein 1	-1.04	0.03
ACKR1	atypical chemokine receptor 1 (Duffy blood group)	-1.04	0.02
SESN2	sestrin 2	-1.03	0.02
PTGER2	prostaglandin E receptor 2	-1.01	0.00
gene:ENSSSCG00000014909		-1.01	0.05
IFIT1	interferon induced protein with tetratricopeptide repeats 1	-0.96	0.00
PLVAP	plasmalemma vesicle associated protein	-0.95	0.00
gene:ENSSSCG00000036785		-0.94	0.01
ITGA4	integrin subunit alpha 4	-0.94	0.03
gene:ENSSSCG00000036675		-0.93	0.05
CMPK2	cytidine/uridine monophosphate kinase 2	-0.93	0.03
DNAH8	dynein axonemal heavy chain 8	-0.92	0.04
SHD	Src homology 2 domain containing transforming protein D	-0.91	0.04
GXYLT1	glucoside xylosyltransferase 1	-0.89	0.01
gene:ENSSSCG00000037377		-0.89	0.04
THAP1	THAP domain containing 1	-0.89	0.00
TMEM88	transmembrane protein 88	-0.85	0.00
MMRN1	multimerin 1	-0.85	0.02
FBF1	Fas binding factor 1	-0.84	0.05
STK17B	serine/threonine kinase 17b	-0.84	0.02

FHL5	four and a half LIM domains 5	-0.84	0.00
ZSCAN4	zinc finger and SCAN domain containing 4	-0.83	0.01
RAC2	Rac family small GTPase 2	-0.81	0.05
RAB27A	RAB27A, member RAS oncogene family	-0.81	0.03
TTC38	tetratricopeptide repeat domain 38	-0.80	0.05
TBC1D8B	TBC1 domain family member 8B	-0.80	0.02
FABP4	fatty acid binding protein 4	-0.79	0.00
NLRC5	NLR family CARD domain containing 5	-0.79	0.02
gene:ENSSSCG00000022195		-0.79	0.04
gene:ENSSSCG00000017608		-0.75	0.04
SLC39A8	solute carrier family 39 member 8	-0.75	0.02
RNF128	ring finger protein 128, E3 ubiquitin protein ligase	-0.75	0.00
DHRS7C	dehydrogenase/reductase 7C	-0.74	0.03
gene:ENSSSCG00000001396		-0.74	0.02
SERHL2	serine hydrolase like 2	-0.73	0.00
TPMT	thiopurine S-methyltransferase	-0.73	0.03
CTSW	cathepsin W	-0.72	0.02
PTPRC	protein tyrosine phosphatase, receptor type C	-0.72	0.01
MDK	midkine	-0.72	0.01
FAM105A	family with sequence similarity 105 member A	-0.70	0.04
L1CAM	L1 cell adhesion molecule	-0.68	0.05
RAPGEF3	Rap guanine nucleotide exchange factor 3	-0.68	0.01
CORO1A	coronin 1A	-0.68	0.05
BEND6	BEN domain containing 6	-0.67	0.04
MAP1B	microtubule associated protein 1B	-0.66	0.00
RNF19B	ring finger protein 19B	-0.66	0.00
CYTIP	cytohesin 1 interacting protein	-0.65	0.02
ZNF649	zinc finger protein 649	-0.65	0.05
ZNF436	zinc finger protein 436	-0.64	0.01
ALKBH1	alkB homolog 1, histone H2A dioxygenase	-0.64	0.04

PFKP	phosphofructokinase, platelet	-0.63	0.00
GTSE1	G2 and S-phase expressed 1	-0.62	0.02
MX2	MX dynamin like GTPase 2	-0.62	0.02
F8	coagulation factor VIII	-0.62	0.03
CA4	carbonic anhydrase 4	-0.62	0.00
ZCCHC2	zinc finger CCHC-type containing 2	-0.61	0.02
KLHL41	kelch like family member 41	-0.61	0.01
UBXN7	UBX domain protein 7	-0.59	0.03
gene:ENSSSCG00000035293		-0.59	0.01
TRAPPC4	trafficking protein particle complex 4	-0.59	0.04

Supplemental Table S5.

Top Involved Canonical Pathways and Diseases and Functions of Differentially Expressed Genes (DEGs) Between RVOT-VPC and Sham Control Groups

Location	Top Canonical Pathways and Diseases and Functions	Involved DEGs	P value
**Septum***	Actin Cytoskeleton Signaling	FGF16,RAC2,PAK1,CYFIP2,FN1,PAK3,DIAPH3,PIK3R6,FGFR2, MYLK,ACTG2,ITGA4,IQGAP3	0.006309573
	Signaling by Rho Family GTPases	MAP3K12,PAK1,CDH12,GNB3,PAK3,DIAPH3,CDH24,PIK3R6, FGFR2,MYLK,ACTG2,ITGA4	0.036307805
	RhoGDI Signaling	PAK1,CDH12,GNB3,PAK3,CREBBP,CDH24,ARHGAP4,ACTG2,ITGA4,EP300	0.019054607
	ILK Signaling	ITGB2,FBLIM1,FN1,CREBBP,PIK3R6,FGFR2,KRT18,ACTG2,MMP9,EP300	0.033113112
	LXR/RXR Activation	LYZ,SERPINF1,ARG2,S100A8,MMP9,TNFRSF11B,AGT	0.042657952
	Cardiac Hypertrophy Signaling	ADRA2B,MAP3K12,CACNA1S,PLCB2,GNB3,IGF1,CREBBP,PIK3R6 ,FGFR2,EP300,ADRB2	0.045708819
**Free wall†**	Actin Cytoskeleton Signaling	RAC2,FGF16,CYFIP2,MYL4,ACTG2,MYH11,PDGFD,MYL1,ITGA4,FGF13	0.033113112
	Signaling by Rho Family GTPases	MAP3K9,FOS,CDH12,GNB3,ARHGEF16,CDH24,MYL4,ACTG2,CDH16 ,MYL1,ITGA4	0.031622777
	RhoGDI Signaling	CDH12,GNB3,ARHGEF16,CDH24,MYL4,ACTG2,CDH16,MYL1,ITGA4	0.020892961
	ILK Signaling	FOS,FBLIM1,MYL4,KRT18,ACTG2,MYH11,PTGS2,PPP2R1B,MMP9,MYL1	0.014454398
	LXR/RXR Activation	APOE,LCAT,IL1RL2,CCL2,SERPINF1,ARG2,S100A8,IL6,PTGS2,MMP9	0.000501187
	Cardiac Hypertrophy Signaling	MAP3K9,CACNA1S,GNB3,ADCY5,IL6R,MYL4,IL6,MYL1,ADRA1D,ADRB2	0.043651583

*: The canonical pathways/diseases/functions derived from 634 differentially expressed genes of left ventricular septum.

†: The canonical pathways/diseases/functions derived from 557 differentially expressed genes of left ventricular free wall.

Supplemental Figure S1.

Hemodynamics obtained before creating RVOT bigeminy VPC in the pig models and before sacrifice at 6-month follow-up. There were no significant differences in pressures (P) in the ascending aorta (AO), left ventricle (LV), pulmonary artery (PA), right ventricle (RV) and right atrium (RA) between RVOT-VPC group and sham control group.

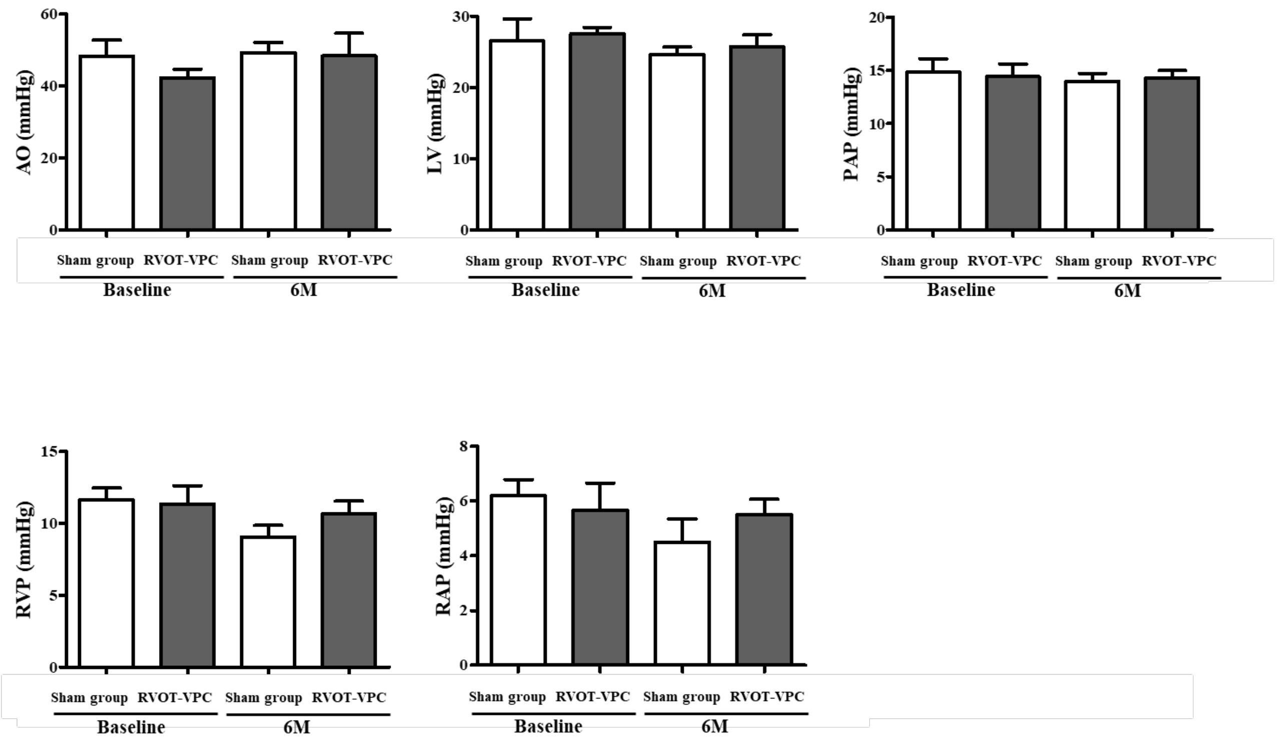

Supplemental Figure S2.

(A) Unsupervised hierarchical clustering of RNA next generation sequencing expression values in the left ventricular septum between RVOT-VPC group and sham control group. A total of 634 genes were identified, of which 269 of these genes were differentially upregulated and 365 were differentially downregulated. **(B) Unsupervised hierarchical clustering of RNA next generation sequencing expression values in the left ventricular free wall between RVOT-VPC group and sham control group.** A total of 557 genes were identified, of which 330 genes were differentially upregulated and 227 genes were differentially downregulated. Bar color indicates mRNA expression level. Red indicates up-regulation; white, no change; blue, down-regulation. Activation z-score analysis method was used to measure activation states of the top involved canonical pathways affected by differentially expressed genes between RVOT-VPC group and sham control group and six pathways were altered at both septum **(C)** and free wall **(D)** of left ventricle. Blue bar indicates negative z-score (inhibition of pathway) while red bar indicates positive z-score (activation of pathway). Activation z-score analysis showed only actin cytoskeleton signaling, RhoGDI signaling, signaling by Rho Family GTPases and ILK Signaling presented z-scores with same activation states in both the left ventricular septum and left ventricular free wall. The ratio of polyline represented the number of differentially expressed genes involved in a pathway divided by the total number of genes in a pathway.

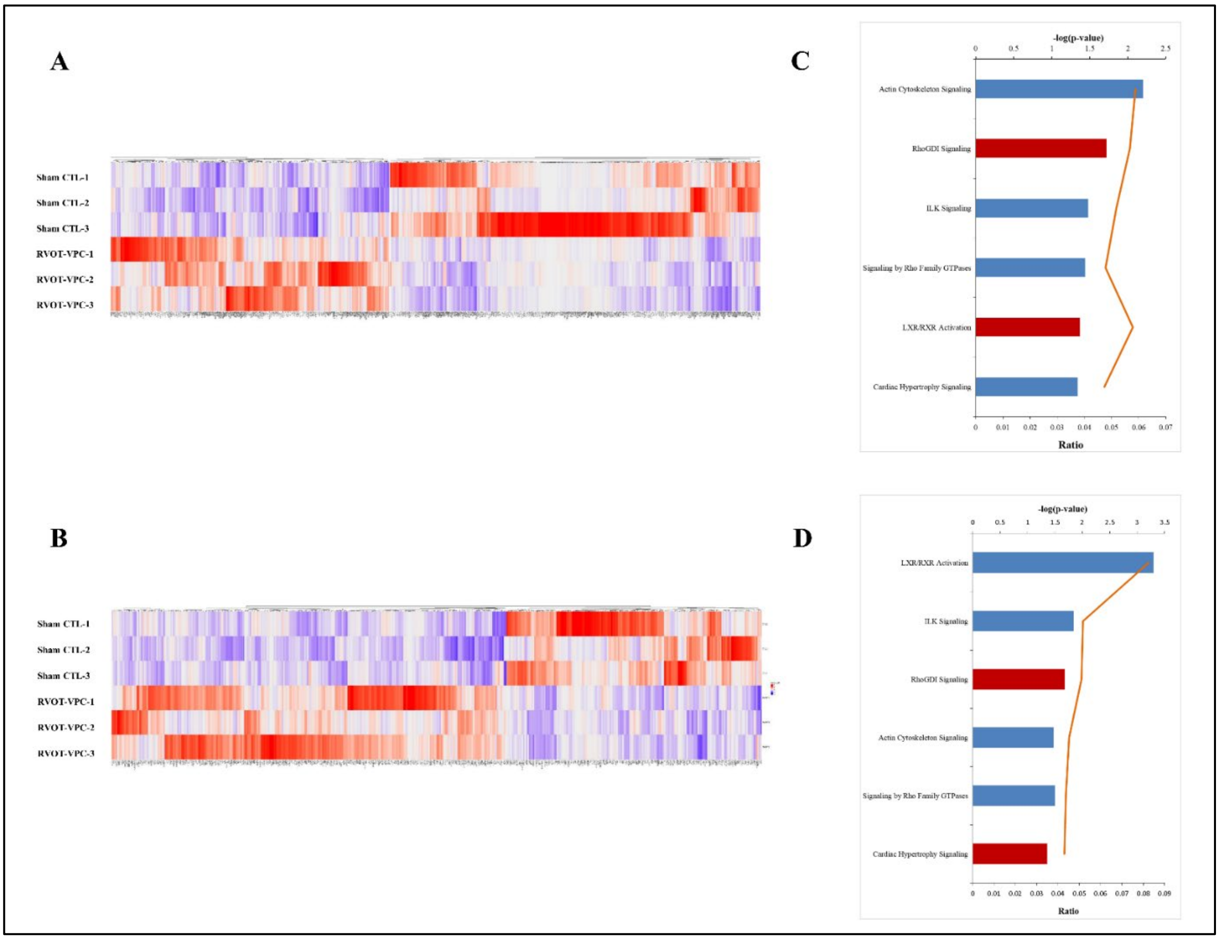
A
B
C
D
Sham CTL-1
Sham CTL-2
Sham CTL-3
RVOT-VPC-1
RVOT-VPC-2
RVOT-VPC-3
-log(p-value)
Actin Cytoskeleton Signaling
RhoGDI Signaling
ILK Signaling
Signaling by Rho Family GTPases
LXR/RXR Activation
Cardiac Hypertrophy Signaling
Ratio

Supplemental Figure S3.

Flowchart of the creation of a right ventricular outflow tract (RVOT) bigeminy ventricular premature contraction (VPC) pig model, echocardiographic examination, next generation sequencing (NGS) analysis, functional enrichment analysis and genes, proteins and histochemical studies. Echocardiograms were performed twice, first within 1 week before creating RVOT PVC model and then within 1 week before all pigs were sacrificed at the 6-month follow-up in the RVOT VPC and sham control groups. In both groups, ventricular sensing (A port) lead was placed at the RV free wall and ventricular pacing (V port) lead was placed at the RVOT septum. Myocardial tissues of the septum and free wall of the left ventricle (LV) at the level of the papillary muscle were dissected and collected after sacrifice at the 6-month follow-up in both groups and some tissues were stored in optimum cutting temperature compound (OCT) for immunofluorescence studies. The midmyocardial layer of the myocardial tissues was sent for immunofluorescence, NGS and Western blotting/PCR studies. RA: right atrium; PA: pulmonary artery; Ao: ascending aorta.

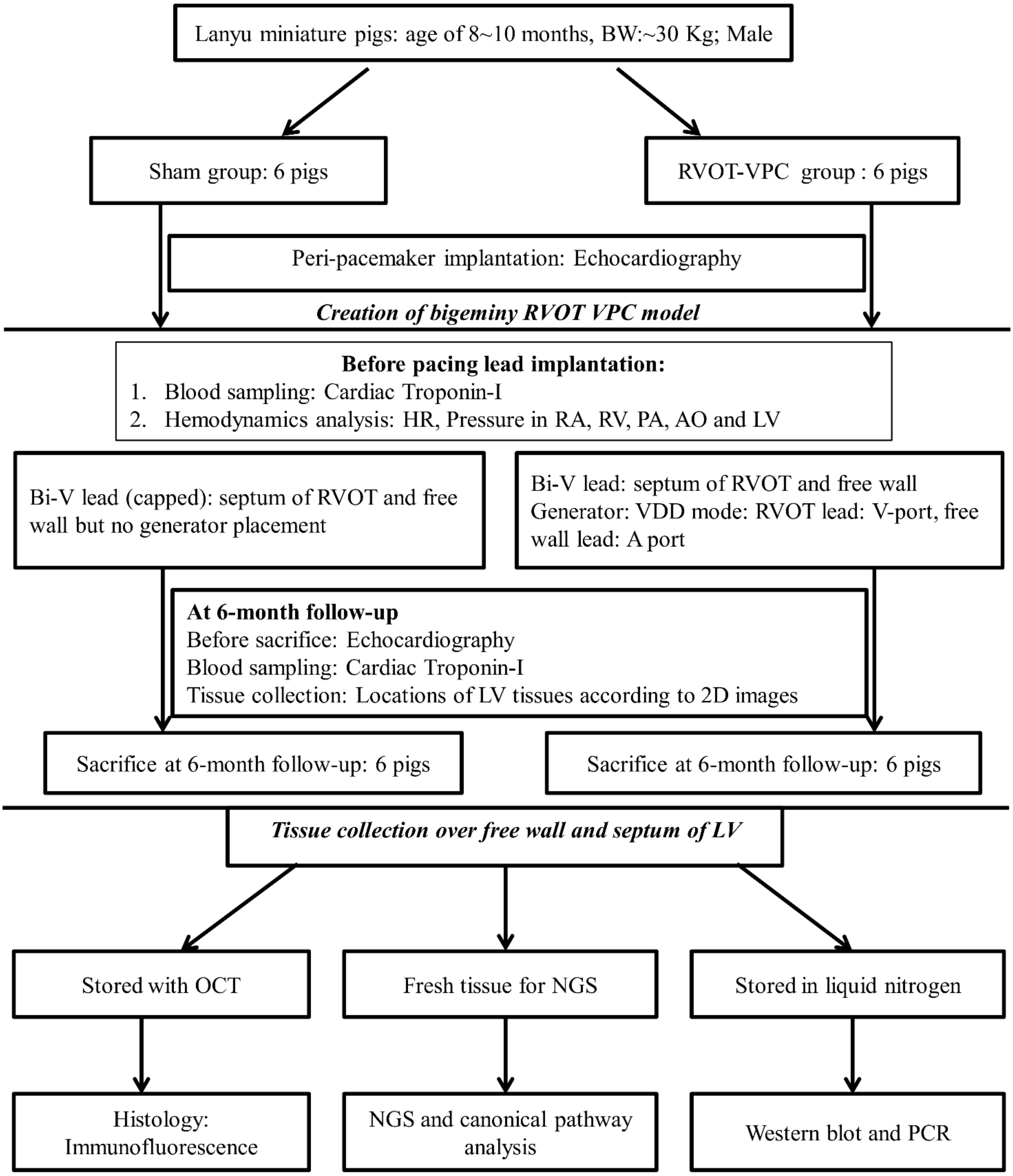
Lanyu miniature pigs: age of 8~10 months, BW:~30 Kg; Male
Sham group: 6 pigs
RVOT-VPC group : 6 pigs
Peri-pacemaker implantation: Echocardiography
Creation of bigeminy RVOT VPC model
Before pacing lead implantation:
1. Blood sampling: Cardiac Troponin-I
2. Hemodynamics analysis: HR, Pressure in RA, RV, PA, AO and LV
Bi-V lead (capped): septum of RVOT and free wall but no generator placement
Bi-V lead: septum of RVOT and free wall
Generator: VDD mode: RVOT lead: V-port, free wall lead: A port
At 6-month follow-up
Before sacrifice: Echocardiography
Blood sampling: Cardiac Troponin-I
Tissue collection: Locations of LV tissues according to 2D images
Sacrifice at 6-month follow-up: 6 pigs
Sacrifice at 6-month follow-up: 6 pigs
Tissue collection over free wall and septum of LV
Stored with OCT
Fresh tissue for NGS
Stored in liquid nitrogen
Histology: Immunofluorescence
NGS and canonical pathway analysis
Western blot and PCR

Supplemental Figure S4.

Locations of sensing/pacing leads on fluoroscopy and at autopsy. The active bipolar lead was inserted into right ventricle and fixed over the free wall of right ventricle, which served as the sense lead and was connected to the A port of pacemaker. Another active bipolar lead was inserted into right ventricle and fixed over the septum of right ventricular outflow tract (RVOT), which served as the pacing lead and was connected to the V port of pacemaker. The locations of leads were presented in a cartoon image **(A),** in gross anatomy **(B)** and on fluoroscopic image in anterior-posterior view **(C)**. The created RVOT bigeminy VPCs showed positive QRS complexes in leads II, III and aVF (red arrows) **(D)**.

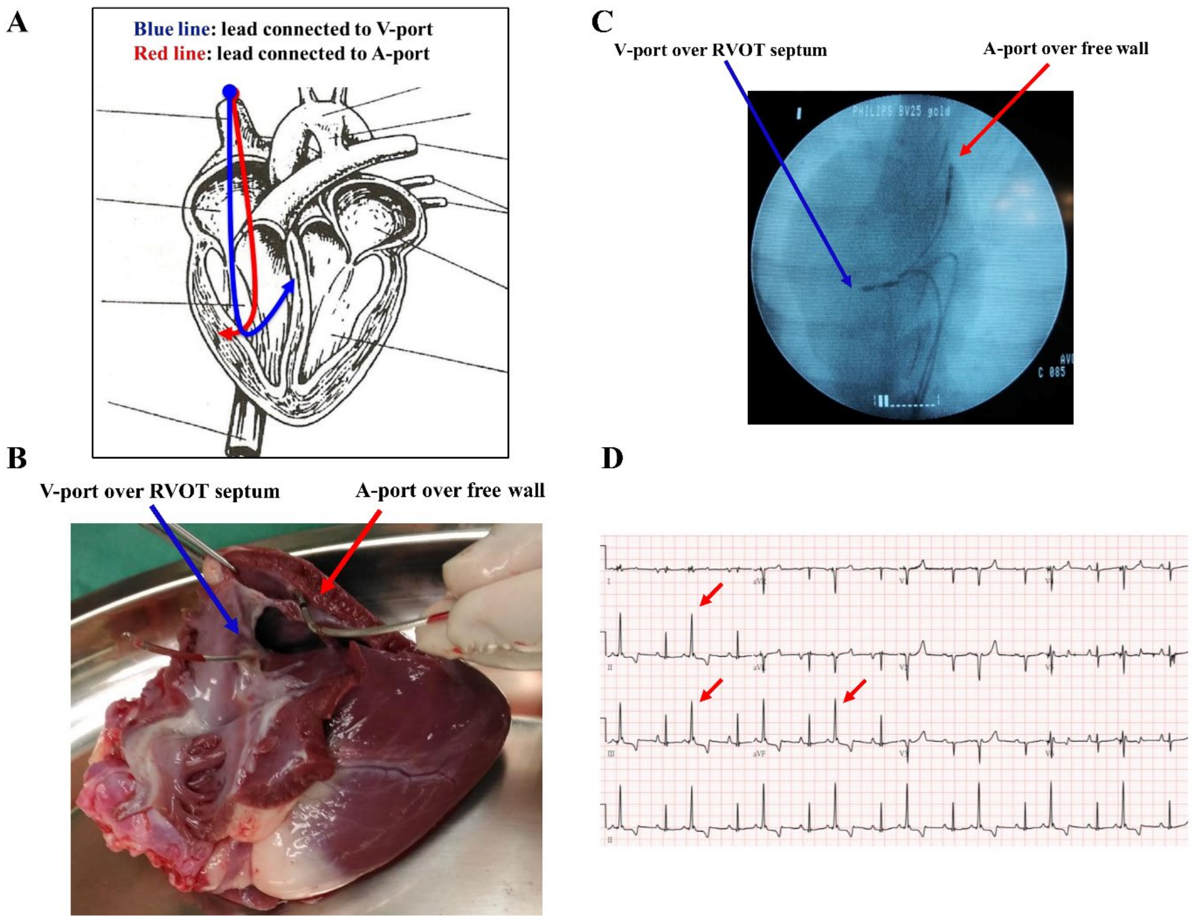

Supplemental Figure S5.

Histogram and percentage of ventricular pacing recorded in generator in 6-month follow-up period. After the sense lead and pacing lead were fixed to right ventricle, the function of pacing/sensing was ensured to work in bigeminy VPC model **(A).** In the 6-month follow up period, the generator was interrogated at 1 week **(B);** 1 month **(C)**; 3 months **(D)** and the time before sacrifice **(E)** to ensure the function of RVOT pacing over >99% pacing to create RVOT bigeminy VPC. Also, in panel **(E)**, we illustrated a12-lead electrocardiogram to show the feature of RVOT bigeminy VPC before sacrifice.

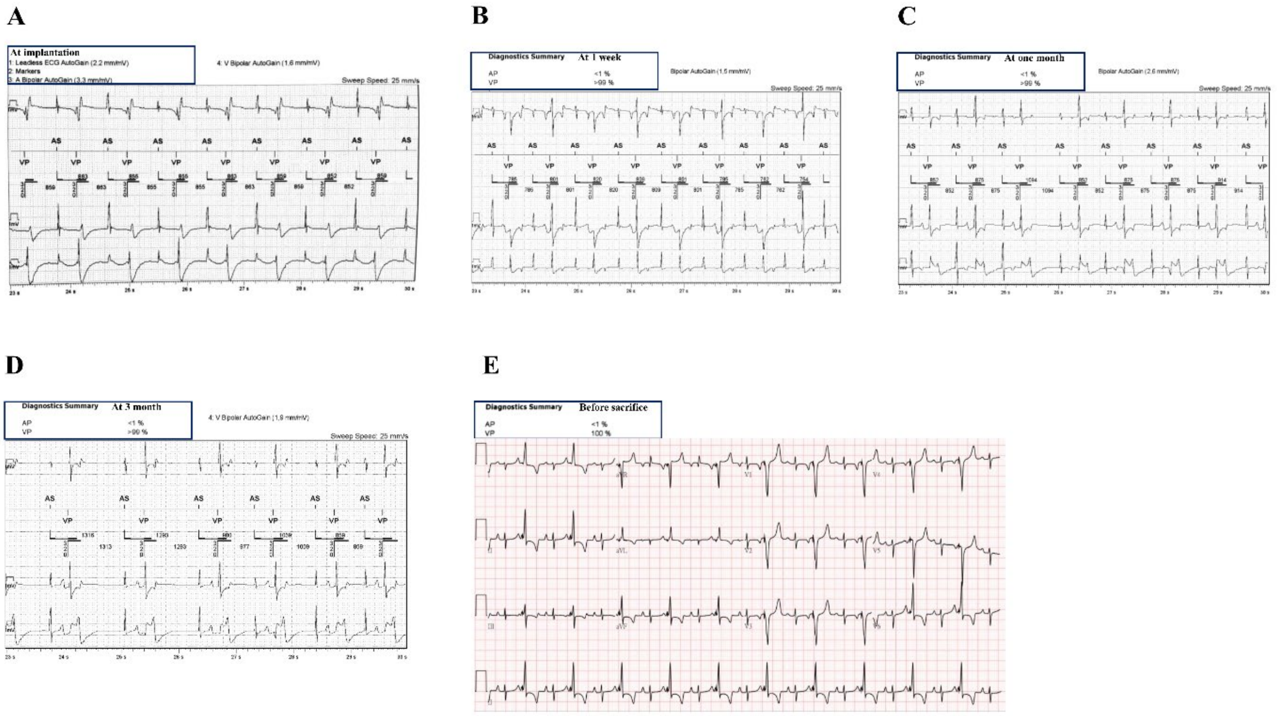

Supplement: Supplementary file 1 [file ijms-22-11244-s001.zip › ijms-1398229-supplementary.pdf]
